# Fault tolerant trust based task scheduler using Harris Hawks optimization and deep reinforcement learning in multi cloud environment

**DOI:** 10.1038/s41598-023-46284-9

**Published:** 2023-11-06

**Authors:** Sudheer Mangalampalli, Ganesh Reddy Karri, Sachi Nandan Mohanty, Shahid Ali, M. Ijaz Khan, Dilsora Abduvalieva, Fuad A. Awwad, Emad A. A. Ismail

**Affiliations:** 1 School of Computer Science and Engineering, VIT-AP University, Amaravati, AP 522237 India; 2 Electronics Engineering, Peking University, Beijing, 100871 China; 3 Department of Mathematics and Statistics, Riphah International University I-14, Islamabad, 44000 Pakistan; 4 Doctor of Philosophy in Pedagogical Sciences, Tashkent State Pedagogical University, Bunyodkor Avenue, 27, 100070 Tashkent, Uzbekistan; 5 Department of Quantitative Analysis, College of Business Administration, King Saud University, P.O. Box 71115, 11587 Riyadh, Saudi Arabia; 6 Department of Mechanical Engineering, Lebanese American University, Beirut 1102-2801, Lebanon

**Keywords:** Biochemistry, Engineering

## Abstract

Cloud Computing model provides on demand delivery of seamless services to customers around the world yet single point of failures occurs in cloud model due to improper assignment of tasks to precise virtual machines which leads to increase in rate of failures which effects SLA based trust parameters (Availability, success rate, turnaround efficiency) upon which impacts trust on cloud provider. In this paper, we proposed a task scheduling algorithm which captures priorities of all tasks, virtual resources from task manager which comes onto cloud application console are fed to task scheduler which takes scheduling decisions based on hybridization of both Harris hawk optimization and ML based reinforcement algorithms to enhance the scheduling process. Task scheduling in this research performed in two phases i.e. Task selection and task mapping phases. In task selection phase, all incoming priorities of tasks, VMs are captured and generates schedules using Harris hawks optimization. In task mapping phase, generated schedules are optimized using a DQN model which is based on deep reinforcement learning. In this research, we used multi cloud environment to tackle availability of VMs if there is an increase in upcoming tasks dynamically and migrate tasks to one cloud to another to mitigate migration time. Extensive simulations are conducted in Cloudsim and workload generated by fabricated datasets and realtime synthetic workloads from NASA, HPC2N are used to check efficacy of our proposed scheduler (FTTHDRL). It compared against existing task schedulers i.e. MOABCQ, RATS-HM, AINN-BPSO approaches and our proposed FTTHDRL outperforms existing mechanisms by minimizing rate of failures, resource cost, improved SLA based trust parameters.

## Introduction

Cloud Computing is one of the rapid growing paradigm in IT industry renders seamless services to its users on demandly based on requirement of user’s application. Applications to be deployed in cloud paradigm are of different types and they require different computing, storage and network capacities. All these resources can be provisioned by cloud provider by using different types of services virtually as infrastructure, platform and as software to users as and when they required with different pricing models^1^. Every cloud user may not require same cloud deployment model for their application. Therefore, a tailor made customized models are available the cloud users and they are public, private, hybrid deployment models^2^. Rendering of these services to all users with different customized deployment models for different pricing models is a challenge for cloud vendor may have different customers around the world and to schedule and allocate all the requests coming from various heterogeneous resources and to allocate different types of requests to various virtual resources to compute in an effective manner without human intervention is a challenging scenario. Therefore, task scheduling plays a crucial role in cloud computing paradigm^3^. Task scheduling is defined as allocating all the incoming tasks to virtual resources resided in datacentres considered in this paradigm. It is a challenging problem in this paradigm as variety of requests from heterogeneous resources comes to cloud application console where the scheduler need to look up all those requests and it should assign it to an appropriate suitable VM which can process this request. Many of existing authors proposed various task scheduling algorithms such as MOABCQ^4^, RATSHM^5^, AINN-BPSO^6^ which are modelled based on metaheuristic approaches but still these approaches are not focused on failure rate, resource cost, SLA based trust parameters. Ineffective scheduling of tasks leads to increase in delay of processing tasks thereby increase in makespan, resource costs, execution time, energy consumption. It effects various parameters and thereby it effects cloud provider’s quality of service and thereby it violates SLA therefore trust on cloud provider also be decreased. Many Task scheduling algorithms developed by various authors using nature inspired, metaheuristic approaches as task scheduling in this paradigm is highly dynamic and it is of type NP-hard problem which cannot give solution in specific polynomial amount of time and scheduling in cloud is also the same type it is difficult to schedule variety of heterogeneous dynamic tasks on to VMs as it is difficult to predict number of tasks to come onto cloud console. When a task is not properly scheduled onto a VM by considering parameters i.e. run time processing capacity, length of task then task execution process may get delayed which impacts makespan and in some other cases task may fails due to improper assignment of VM there by rate of failures will be increased. When rate of failures increased there is a chance of impact on violation of SLA which impacts both Quality of service and trust on the cloud provider. Trust on the cloud provider depends mainly on success rate of VM, Availability of VM, turnaround efficiency of tasks. For improvement of availability of resource and to minimize resource cost we used multi cloud model and migrate tasks based on availability of resources in the corresponding resource where VMs are available by minimizing resource cost. Therefore, in this research, to minimize rate of failures and to increase trust on cloud provider we developed a task scheduler which schedules tasks in two phases i.e. selection of tasks, mapping of tasks on suitable VMs. In initial phase, tasks for scheduling is done based on all priorities of tasks, VMs collected from task manager and fed to scheduler which generates schedules with the help of Harris Hawks optimization and generated schedules are optimized using a reinforcement learning based model i.e. DQN model that minimizes makespan, rate of failures, resource cost and improving SLA based parameters.

### Motivation and contributions

Task scheduling in cloud paradigm poses challenges to cloud provider as it is difficult to map tasks with different run time capacities to precise VMs. This is a challenge in cloud paradigm and improper mapping or assigning of tasks to VMs effects the QoS of cloud provider. It directly effects makespan, turnaround efficiency of tasks by delaying task execution on VMs which leads to decay of quality service. In some cases, due to ineffective mapping of tasks by scheduler i.e. if size of task or runtime processing capacity is not matched with the VM capacity then there is a chance of failure occurs in that VM. Therefore, rate of failures can also be an effected parameter for ineffective scheduling. Another parameter to be effected in cloud computing paradigm is Availability of VMs as if a task is assigned to VM and if that resource is not available at that instance of time then it directly effects the task execution by making that task to be failed. Success rate of VM effects QoS of cloud provider as if the task assignment is not done accurately onto a VM then it directly effects quality of service, SLA violations. The above reasons motivated us to take up this research while mapping tasks to accurate VMs by considering priorities of Tasks, VMs using both Harris Hawks for selecting tasks and scheduling them and DQN model to optimize generated schedules while addressing makespan, resource cost, SLA based trust parameters.

Highlights of our manuscript are indicated as below.

- A Fault tolerant based task scheduling algorithm(FTTHDRL) for multi-cloud environment.
- This task scheduler modelled using hybrid approach Harris hawk optimization and a DQN model based on deep reinforcement learning.
- Scheduling of tasks to precised VMs modelled in two phases i.e. task selection and generation of schedules using Harris Hawks optimization. In task mapping phase optimization of generated schedules designed by using DQN model which works based on Deep reinforcement learning.
- Extensive simulations are conducted on Cloudsim with fabricated and realtime computing worklgs from HPC2N, NASA.
- Proposed FTTHDRL evaluated against existing approaches i.e. RATS-HM, MOABCQ, AINN-BPSO and evaluated parameters makespan, resource cost, rate of failures, SLA based trust parameters in multi cloud environment.

Rest of the manuscript is organized as indicated below. Section “Existing related works” discusses existing related works, Section “Fault tolerant trust aware task scheduling using Harris Hawks and DRL in multi cloud environment” discusses Proposed architecture of FTTHDRL, Section “Simulation and results” discusses methodology used for our proposed approach i.e. Harris Hawks optimization, DQN model, Section “Conclusion and future works” discusses Simulation and Results. Finally Section 6 discusses Conclusion and Future works.

## Existing related works

This section clearly discusses about various existing task schedulers which addresses different parameters and techniques they used to develop schedulers in cloud computing paradigm. In^7^, authors focused on scheduling tasks effectively by minimizing makespan, execution time on VMs. They improved differential evolution approach to a hybrid level by incrementing scaling factors to enhance exploration, exploitation in the searching process of a solution in search space. All experimentation conducted on Cloudsim. HDE compared against state of art algorithms SMA, EO, GWO, FCFS, RR to check effectiveness of proposed HDE. Results proved that HDE dominant with respect to execution time, makespan. Penalty function which is related to SLA violations in cloud computing plays a major role in this paradigm. Authors in^8^, formulated adaptive task scheduling mechanism which addressed penalty function as a parameter to adjust scheduling process dynamically as per SLA made by cloud provider. Symbiotic organisms search is improved to enhance scheduling process to adapt to search space as and when more workload generated it schedules workload to VMs. This simulation conducted on Cloudsim with random workload and results proved that penalty related to SLA violation minimized with ABFSOS. Authors in^9^ formulated a task scheduling mechanism which uses improved version of MVO approach by adjusting average position of solution in scheduling. These simulations are conducted on Cloudsim and evaluated parameters i.e. execution time, throughput, VM processing Power. It evaluated over existing algorithms MVO, mMVO and proposed IMOMVO reveals that it improves above said parameters while compared with existing approaches. Authors in^10^ developed multi objective task scheduling model addressed the parameters consumption of energy, makespan. A hybrid approach was used to model task scheduler. This model works based on combining Whale optimization, differential evolution techniques for better enhancement of scheduling process in cloud paradigm. Simulations performed extensively in developing this scheduler which takes standard realtime datasets as input to algorithm i.e. curie workloads, HPC2N by varying different number of iterations. Results revealed that h-DEWOA dominates other non-differential approaches by minimizing both energy consumption, makespan. A multi objective scheduling mechanism developed to tackle parallel workloads in cloud environment. This approach aims at makespan, throughput. This mechanism modelled by using hybrid BAT approach to explore near optimal solution in search space. Real time parallel workload given as an input to algorithm and simulations are conducted on Cloudsim. Performance of hybrid BAT evaluated over classical BAT and other metaheuristic approaches. Evaluated results evident that it outperforms existing mechanisms for specified parameters. For the benefit of cloud user and service provider authors in^12^ proposed a task scheduling strategy which provides Quality of service and improves resource utilization. It was modelled using both IQSSA, QSSGWA algorithms which were inspired based on Quantum computing, Salp swarm algorithms. All simulations are conducted on MATALAB. Both IQSSA, QSSGWA tested against more than 10 benchmark functions to evaluate convergence of proposed approach. It evaluated against existing approaches SSA, GWO algorithms. These approaches shown huge impact over state of art algorithms for above mentioned parameters. Degree of imbalance for tasks is one of the major concern in cloud computing as they rush towards application console. This parameter efficiently tackled by authors in^13^. Initially they used WOA to explore its ability in local search process while horse optimization is used as global search to fine tune convergence rate. It was compared to baseline mechanisms PSO, GWO, WOA approaches to address makespan, degree of imbalance. In^14^, authors developed job scheduling mechanism in Fog computing i.e. DOLSSO. It was modelled based on opposition learning social spider optimization combined with a reinforced mechanism which was implemented on Ifogsim. Initially, schedules generation was populated by OLSSO and optimization of those schedules were done by reinforcement strategy used in the approach. Workflows used in simulation are generated randomly in Ifogsim. It was ratified against state of art approaches and results shown that DOLSSO dominates other approaches for utilization of CPU, consumption of energy. Resource cost is also a crucial parameter which effects scheduling impact in cloud computing paradigm. It was discussed by authors in^15^. They developed a task scheduling mechanism which consists of both cuckoo search and harmony search to tackle scheduling problem in cloud computing. CS acts as local search to explore solution space while HS used as global search to explore solution space. Simulations are conducted on Cloudsim tool. It was compared over CS, HS, CGSA approaches. Finally from generated results of CSHA it proved that resource cost, penalty is minimized over existing approaches. Authors in^16^ also focused on total execution cost in Fog computing that effects scheduling. They developed a task scheduling mechanism which is based on hyper heuristic approach which tunes convergence of solutions. It evaluated against baseline metaheuristic approaches and entire simulation conducted on Ifogsim. Results shown the effectiveness of HHS on other approaches while minimizing execution cost, latency, execution time. A Job scheduling strategy developed in^17^ to find a best possible VM to execute tasks which are highly dynamic in IOT applications. This mechanism was developed in two stages. In first stage, all the nodes are clustered together and grouped them together and trained with different levels of utilization. In second stage, SSA combined with DE used to optimize degree of imbalance, throughput, consumption of energy. Power consumption in datacentres effects cloud provider as if proper scheduler is not used to execute tasks, therefore there is an increase in consumption of power in cloud paradigm which effects cloud provider directly but it also effects customer because they need to pay extra pricing for services they consume. Authors in^18^ developed a scheduler which tackles with power consumption, execution cost, runtime. It was modelled using NSGA-III which considers an adaptive fitness function to adjust and schedule tasks to VMs appropriately. From results, it proved that NSGA-III dominated other approaches in terms of power consumption, cost, Runtime. Resource allocation optimization in cloud paradigm is challenging issue as mapping tasks to VMs is a challenge in cloud paradigm as it is a NP hard problem. For this to happen, authors in^19^ proposed task scheduler which developed by combining GTO, RSO. This approach initially predicts features in upcoming tasks by extracting them using PCA. These features are fed to HMEERA which allocates tasks to virtual resources by optimizing them using GTO, RSO approaches. It was compared over existing approaches and results shown dominance of HMEERA in view of response time, waiting time. Execution time, Cost are prominent concerns for task scheduling in cloud model. These parameters are addressed and tackled in^20^ by using hybrid technique HWOA-MBA. It was developed by enhancing RDWOA by tuning mutation of Bee’s algorithm. It was simulated on Cloudsim and ratified against MALO, IWC, BA-ABC. Results shown that proposed HWOA-MBA dominated over state of art algorithms for task completion time, execution time. In^21^, authors developed a task scheduler which improves makespan in cloud computing paradigm. This was developed by using IPSO which is an improved version of PSO by segregating particles as ordinary, local best particles which converges towards solution fast when compared with classical PSO. IPSO ratified over CEC 2017 benchmark and results shown huge impact over state of art algorithms for makespan, load balancing. Authors in^22^ proposed an AI based scheduling technique to precisely map tasks to VMs while addressing rate of task completion, energy consumption. It was modelled by extending GGCN by adding a recurrent unit to it. Simulation results of HunterPlus shown it was greatly minimizes consumption of energy by 17%, improves rate of task completion by 10%. Resource cost, makespan are addressed by authors in^23^ by formulating a task scheduler for cloud paradigm which is a hybridized approach by integrating adaptive weight strategy to ACO algorithm. It was compared over ACO, Min-Min, MTF-BPSO algorithms. Results of HWACO shown improvement in makespan, cost over compared approaches. In^24^ bio inspired task scheduling paradigm which deals with cost saving of resources. It was designed by sea gull optimization technique which adapts to cloud environment. It compared over CJS, MSDE, FUGE approaches and finally results evident that cost of virtual resources, consumption of energy minimized with SOATS. In^25^, task scheduling algorithm which optimizes quality of service parameters concerned with cloud computing. It was modelled as a hybridized approach by combining rider optimization with cuckoo search to get adapted to dynamic nature of cloud paradigm. It was implemented on Cloudsim tool. RCOA ratified against COA, Rider algorithm, PSO. RCOA shown huge impact while minimizing makespan. Multi objective scheduling technique developed by authors in^26^ using QOGSHO by combining QOBL, SHO by designing an adaptive fitness function to evaluate SLA Violation, makespan, resource utilization. This experimentation conducted on a customized cloud environment. Services for mobile computations are restricted as it is difficult to assign resources to applications in mobiles. Therefore, offloading is a technique where intensive computations are migrated and offloaded to cloud servers for computing tasks. This offloading process designed by authors in^27^ using African wild dog algorithm based on hunting cooperation behaviour of wild dogs. This entire simulation process conducted on Cloudsim. AWDA evaluated over existing approaches. Results of AWDA dominated other state of art algorithms by minimizing cost, delay time, energy. User satisfaction to gain trust over cloud provider is a serious concern in terms of business of cloud provider. Authors in^28^ hybridized PSO, GA algorithms to handle the concerns related to user satisfaction, processing efficiency. Cloudsim tool is used to conduct simulations. All simulations are conducted with uniform simulation settings for all the other algorithms to which PGSAO is ratified against them. Simulation results shown high impact over against existing state of art approaches by improving user satisfaction. Concurrent tasks are difficult to schedule tasks in VMs resided in Physical nodes. In datacentres minimizing energy consumption is a huge challenge in datacentres as tasks are raised from various resources to cloud application console^29^. In the first phase, OBL, PSO are integrated with WOA algorithm to enhance performance of algorithm. In second phase, OBL, PSO optimizes exploration and minimizes energy consumption, makespan over existing state of art algorithms. In^30^, task scheduling algorithm is formulated to address multiple objectives availability, success rate, makespan, turnaround efficiency. ICOATS is proposed quality of service scheduling algorithm which concerned with length of task, priorities. It was simulated on Cloudsim. It ratified over state of art approaches. Results of simulations dominated in terms of above mentioned parameters. Task scheduling algorithm with multiple objectives developed in^31^ to address makespan, execution cost, utilization of resources in integrated cloud-fog computing model. This approach modelled using IJFA by considering with variations in sizes of tasks, task speed, capacity of VMs in cloud-fog environment. Ifogsim was used as a simulation environment and ratified over state of art approaches to check the efficacy of IJFA.

From the above section “Existing related works” and Table 1 it is clearly observed that earlier authors who formulated task scheduling algorithms addressed parameters execution time, cost, utilization of resources, makespan, consumption of energy. They haven’t addressed SLA based trust parameters in a multi cloud environment with inclusion of task, VM priorities. This approach modelled by using hybridization of Harris hawks Optimization algorithm(HHOA), DQN model which is a reinforcement learning based technique to optimize generated schedules which minimize makespan, resource cost, rate of failures and improves SLA based trust parameters.

**Table 1:** Parameters and technique used in various task scheduling algorithms in cloud computing.

References	Methodology used	Addressed parameters
^7^	HDE	Makespan, Execution time
^8^	ABFSOS	Penalty function
^9^	IMOMVO	Execution time, throughput, VM Processing Power
^10^	h-DEWOA	Energy Consumption, makespan
^11^	Hybrid BAT	Makespan, throughput
^12^	IQSSA, QSSGWA	Quality of Service, Resource utilization, rate of SLA Violation
^13^	IWHOLF-TSC	Makespan, degree of imbalance, resource utilization
^14^	DOLSSO	CPU Utilization, Energy Consumption
^15^	CHSA	Cost, memory usage, energy consumption, penalty
^16^	HHS	Consumption of energy, total execution cost, total execution time, latency
^17^	CSSA-DE	Throughput, degree of imbalance, resource utilization, consumption of energy
^18^	NSGA-III	Runtime, power consumption, cost
^19^	HMEERA	Waiting time, response time, load balancing
^20^	HWOA-MBA	Task Completion time, execution time
^21^	IPSO	Makespan, load balancing
^22^	Hunterplus	Consumption of energy, rate of task completion
^23^	HWACO	Makespan, cost
^24^	SOATS	Cost, consumption of energy
^25^	RCOA	Makespan, energy consumption
^26^	QOGSHO	Makespan, resource utilization, SLA Violation
^27^	AWDA	Delay time, cost, energy
^28^	PGSAO	User satisfaction, Processing efficiency
^29^	OWPSO	Makespan, energy consumption
^30^	ICOATS	Makespan, turnaround efficiency, availability, success rate
^31^	IJFA	Makespan, execution cost, resource utilization
^32^	PFA	Total execution time, cost, resource utilization
^33^	Hybrid FPA	Makespan, degree of imbalance
^34^	Hybrid Lion-GA	Turnaround time, resource usage
^35^	G-SOS	Makespan
^36^	HMOA	Execution time

## Fault tolerant trust aware task scheduling using Harris Hawks and DRL in multi cloud environment

This section discusses overall system architecture and mathematical modelling used in Fault tolerant trust aware model developed for multi cloud environment modelled by hybridizing Harris Hawks Optimization algorithm(HHOA) and DQN models which is used to check availability for multiple cloud environments to map tasks to corresponding VMs to minimize rate of failures, resource cost in this model. The subsection “FTTHDRL Problem definition and system architecture” discusses FTTHDRL mathematical modelling, problem formulation.

### FTTHDRL problem definition and system architecture

In this subsection, we precisely formulated problem definition for FTTHDRL(Fault tolerant trust aware Harris Hawk and Deep reinforcement Learning) based system architecture. Assume that we have $i$ number of tasks represented as $\{t{a}_{1},t{a}_{2},t{a}_{3}\dots .,t{a}_{i}\}$, $j$ number of VMs represented as $\left\{{v}_{1},{v}_{2},\dots {v}_{j}\right\}$, $k$ number of physical nodes represented as $\{p{n}_{1},p{n}_{2},\dots .p{n}_{k}\}$, $l$ number of datacentres represented as $\{{d}_{1},{d}_{2},{d}_{3}\dots .,{d}_{l}\}$. Now problem formulation can be done as $i$ tasks are mapped to $j$ VMs placed in $k$ physical nodes which are placed in $l$ datacentres by considering priorities of both tasks, VMs while tackling all the parameters. Initially, all tasks arises from various resources which have different processing capacities. All these tasks consists of different lengths, runtime capacities. For every task which is coming from user will be submitted to cloud application console which are captured by broker included in the cloud provider module. This broker will keep track of priorities of all tasks. For all the tasks, priorities are calculated based on task length, execution time. This scheduling process considers another priority i.e. VM priority based on electricity cost. All these priorities are to be maintained in a priority queue to be fed to the scheduler module which is integrated with Harris Hawk algorithm and DQN model to tackle parameters rate of failures, resource cost, makespan, SLA based trust parameters. While scheduling each task to a VM based on priorities a task with highest priority should be mapped to a VM with highest priority means that VM resided in a datacentre which run with low electricity cost. If there is no VM suitable for the task available in the datacentre as we are using multi cloud environment it will check for the VM in the another cloud and if it is suitable it migrates tasks to another cloud environment which runs with same priority. In another case, if the task need to be mapped to a VM which scans all the VMs available in multi cloud environment and schedules task to a VM which incurs less resource cost. In the first phase, all priorities captured by task manager and fed to scheduler which generates schedules based on HHA algorithm and then all these generated schedules are optimized using DQN model to tackle above mentioned parameters.

### Mathematical modelling of FTTHDRL

This subsection discusses about mathematical modelling of fault tolerant trust aware task scheduler by hybridization of HHA and DRL based DQN model. In the initial phase to generate schedules calculation of priorities for tasks, VMs to be done. In the below equation, present workload on considered VMs in this architecture are calculated using Eq. (1).

1 $$loa{d}_{{v}_{j}}=\sum loa{d}_{j}$$

where $loa{d}_{{v}_{j}}$ is present running tasks workload on $j$ VMs. All these $j$ VMs are placed in $k$ physical nodes. Present workload on all physical nodes are calculated using Eq. (2).

2 $$loa{d}_{p{n}_{k}}=\frac{loa{d}_{j}}{\sum p{n}_{k}}$$

where $loa{d}_{p{n}_{k}}$ is present running tasks on $k$ physical nodes. For calculation of task priorities processing capacities of VMs to be properly identified and they are represented in Eq. (3).

3 $$Pr{o}_{{v}_{j}}=pr{o}_{n}*pr{o}_{mips}$$

Processing capacity of all VMs are represented using below Eq. (4).

4 $$tota{l}_{pr{o}_{{v}_{j}}}=\sum Pr{o}_{{v}_{j}}$$

Priorities of tasks depends on two components i.e. length of task, runtime or processing capacity of a VM. All incoming length of tasks are calculated and they are represented in Eq. (5).

5 $${ta}_{i}^{len}={ta}_{i}^{mips}*{ta}_{i}^{pro}$$

In the step 5, we identified length of all $i$ tasks considered in our system architecture. Now, Priorities of all incoming tasks are represented by Eq. (6).

6 $${ta}_{i}^{pri}=\frac{{ta}_{i}^{len}}{Pr{o}_{{v}_{j}}}$$

In Eq. (6). after calculation of task priorities, we calculated all $j$ VM priorities to schedule all incoming $i$ tasks to suitable VMs considered in our architecture. In order to map tasks appropriately, VM priorities based on electricity cost at datacentre location is represented by Eq. (7).

7 $$v{m}_{j}^{pri}=\frac{hig{h}_{elecost}}{{d}_{l}elecost}$$

From Eqs. (6) and (7) priorities of tasks, VMs are calculated. These priorities are fed to scheduler in which high prioritized task should map to a high prioritized VM. If high prioritized VM is not available at the current cloud vendor look for the high prioritized VM in the another cloud vendor as we are using multi cloud environment. If high prioritized VMs are not available at both the cloud vendors and then look for a VM which is having next highest priority in any cloud vendor which have less resource cost. Therefore, it is necessary to calculate resource cost in cloud model and it is represented by Eq. (8). It is an important parameter need to be addressed in this model as we said earlier our model is aimed at minimization of resource cost which is an important aspect for both cloud provider and user. It is represented in Eq. (8).

8 $$Re{s}_{cost}= \sum_{j=1}^{v{m}_{j}}\frac{cost \, for \, running t{a}_{i}*Memory \, of \, t{a}_{i}}{{v}_{j}*p{n}_{k}}$$

Makespan is one of the important parameter which should be addressed after identifying resource cost in this model. For any task scheduler in the cloud environment it is important to calculate makespan as it impacts quality of service of cloud provider and other parameters which effects SLA violations and related to trust on the cloud provider. It is represented by Eq. (9).

9 $$ms\left(t{a}_{i}\right)=avai{l}_{i}+ex{e}_{j}$$

In this proposed algorithm, another important objective to address is to minimize rate of failures thus by improving fault tolerance using this scheduler in multi cloud environment. It is represented by Eq. (10).

10 $$Fai{l}_{rate}=\frac{mtbf}{mtbf+mttr}$$

where $mtbf$ represents mean time between failures, $mttr$ represents mean time to repair or restore a node from a failure to restore process. We formulated fault tolerance for all $i$ tasks in this model. Our next objective is to relate fault tolerance with SLA based trust parameters which effects Quality of service of cloud provider. SLA based trust parameters are of three types. They are Availability, Success rate, Turnaround efficiency of VMs. Thus, we calculated availability of $j$ VMs and it is represented using Eq. (11).

11 $$avail\left({v}_{j}\right)=\frac{ac{c}_{t{a}_{i}}}{t{a}_{i}}$$

Another trust based parameter need to be calculated is success rate of VMs. It is represented using Eq. (12). It is defined as rate of successful tasks of $t{a}_{i}$ to submitted number of tasks of $t{a}_{i}$. It is represented by Eq. (12).

12 $$su{c}_{rate}\left({v}_{j}\right)=\frac{succes{s}_{t{a}_{i}}}{submitte{d}_{t{a}_{i}}}$$

Turnaround efficiency of a VM is another parameter which effects trust of cloud provider. It is represented by using Eq. (13).

13 $$tur{n}_{eff}\left({v}_{j}\right)=\frac{es{t}_{tim{e}_{t{a}_{i}}}}{ac{{t}_{time}}_{t{a}_{i}}}$$

After evaluation of all these SLA based trust parameters, trust on cloud provider is represented by Eq. (14).

14 $$trs{t}_{cp}={X}_{1}*avail\left({v}_{j}\right)+{X}_{2}*su{c}_{rate}\left({v}_{j}\right)+{X}_{3}*tur{n}_{eff}\left({v}_{j}\right)$$

where ${X}_{1}, {X}_{2}, {X}_{3}$ are coefficient weights which are helpful to evaluate trust on Cloud provider and this value $\epsilon (\mathrm{0,1})$ and it is captured from^37^. It is calculated using co-variance mechanism The weights in the above equations are considered as ${X}_{1}=0.5,{X}_{2}=0.2, {X}_{3}=0.1$ are considered from^37^.

### FTTHDRL fitness function for schedules generation using Harris Hawk optimization

This subsection clearly presents fitness function for generation of schedules using Harris Hawk optimization. Earlier we said we are using a hybrid approach, thus initially we generate schedules by evaluating fitness function using Eq. (15). and there after we use reward function in DQN model to optimize schedules in the second level while improving above said parameters.

15 $$f\left(x\right)={\upbeta }_{1}*Re{s}_{cost}+{\upbeta }_{2}*ms\left(t{a}_{i}\right)+{\upbeta }_{3}*faul{t}_{tol}+{\upbeta }_{4}*avail\left({v}_{j}\right)+{\upbeta }_{5}*su{c}_{rate}\left({v}_{j}\right)+{\upbeta }_{6*}tur{n}_{eff}\left({v}_{j}\right)$$

where ${\upbeta }_{1}+{\upbeta }_{2}+{\upbeta }_{3}+{\upbeta }_{4}+{\upbeta }_{5}+{\upbeta }_{6}=1$. From Eq. (15). as we discussed earlier in the subsection "FTTHDRL fitness function for schedules generation using Harris Hawk optimization", we evaluate fitness function and then check the values of generated parameters using Harris Hawks algorithm.

### Methodology used in FTTHDRL

This subsection clearly presents the methodology used in proposed FTTHDRL. This algorithm modelled by using hybridizing Harris Hawk algorithm and DQN model which is based on reinforcement learning. The below Section “Harris Hawk Optimization” clearly discusses about different phases of Harris Hawk optimization algorithm.

#### Harris Hawk optimization

In this section, initial methodology of FTTHDRL i.e. Harris hawk optimization from^38^ discussed. It works based on cooperative hunting behaviour of Hawks for prey. In the initial stage, it identifies location of the prey based on whether it is alone or in the group. Position of prey represented using Eq. (16).

16 $$Z\left( {T + 1} \right) = \left\{ {\begin{array}{*{20}c} {R \ge 0.5} & {Z^{{RAND}} \left( T \right) - r_{1} \left| {Z^{{RAND}} \left( T \right) - 2r_{2} Z\left( T \right)} \right|} \\ {R < 0.5} & {\left( {~Z^{{RAB}} \left( T \right) - Z^{m} \left( T \right)} \right) - r_{3} \left( {LowB + r_{4} \left( {UpperB - LowB} \right)} \right)} \\ \end{array} } \right.$$

Equation (17) represents average position of Hawk.

17 $${Z}^{m}\left(T\right)=\frac{1}{y}{\sum }_{a=1}^{y}{Z}_{a}^{m}\left(T\right)$$

Now, we evaluate Prey energy and is represented using Eq. (18).

18 $$ENER=2ENE{R}_{0}\left(1-\frac{T}{Y}\right)$$

From Eq. (18), $ENER$ represents escaping energy of prey, $ENE{R}_{0}$ represents initial escaping energy of prey. It ranges from -1 to 1. When energy of prey is decreasing then it is easy for hawk bird to trace prey and exploit. Hawk birds exploit prey when it moves from exploration to exploitation phase but it depends on probability of escaping of prey from hawk, energy required for prey to escape from hawk bird. Prey hunting by hawk bird totally depends on probability of escaping from hawk i.e. if $prob<0.5$ then prey can be escaped or if $prob\ge 0.5$ then prey can be exploited by hawk. Exploitation according to ^38^ mentioned in two phases i.e. softly encircled around the prey by hawks if $prob\ge 0.5 \&\& \left|ENER\right|\ge 0.5$. It is represented here in the eqns. 19, 20.

19 $$Z\left(T+1\right)=\upmu Z\left(T\right)-ENER\mid B{Z}^{RAB}\left(T\right)-Z\left(T\right)\mid$$

20 $$\upmu Z\left(T\right)={Z}^{RAB}\left(T\right)-Z\left(T\right)$$

Hardly encircled by Hawk birds around the prey if $prob\ge 0.5 \&\& \left|ENER\right|\ge 0.5$. It is represented in Eq. (21).

21 $$Z\left(T+1\right)={Z}^{RAB}\left(T\right)-ENER\mid\upmu Z\left(T\right)|$$

There is an another encircling mechanism for prey by using incremental steps by observing the previous movements of prey. It is known as soft encircling mechanism with incrementing their steps up to the prey’s location and it is represented using Eq. (22).

22 $$X={Z}^{RAB}\left(T\right)-ENER\mid B{Z}^{RAB}\left(T\right)-Z\left(T\right)\mid$$

**Figure 1:**
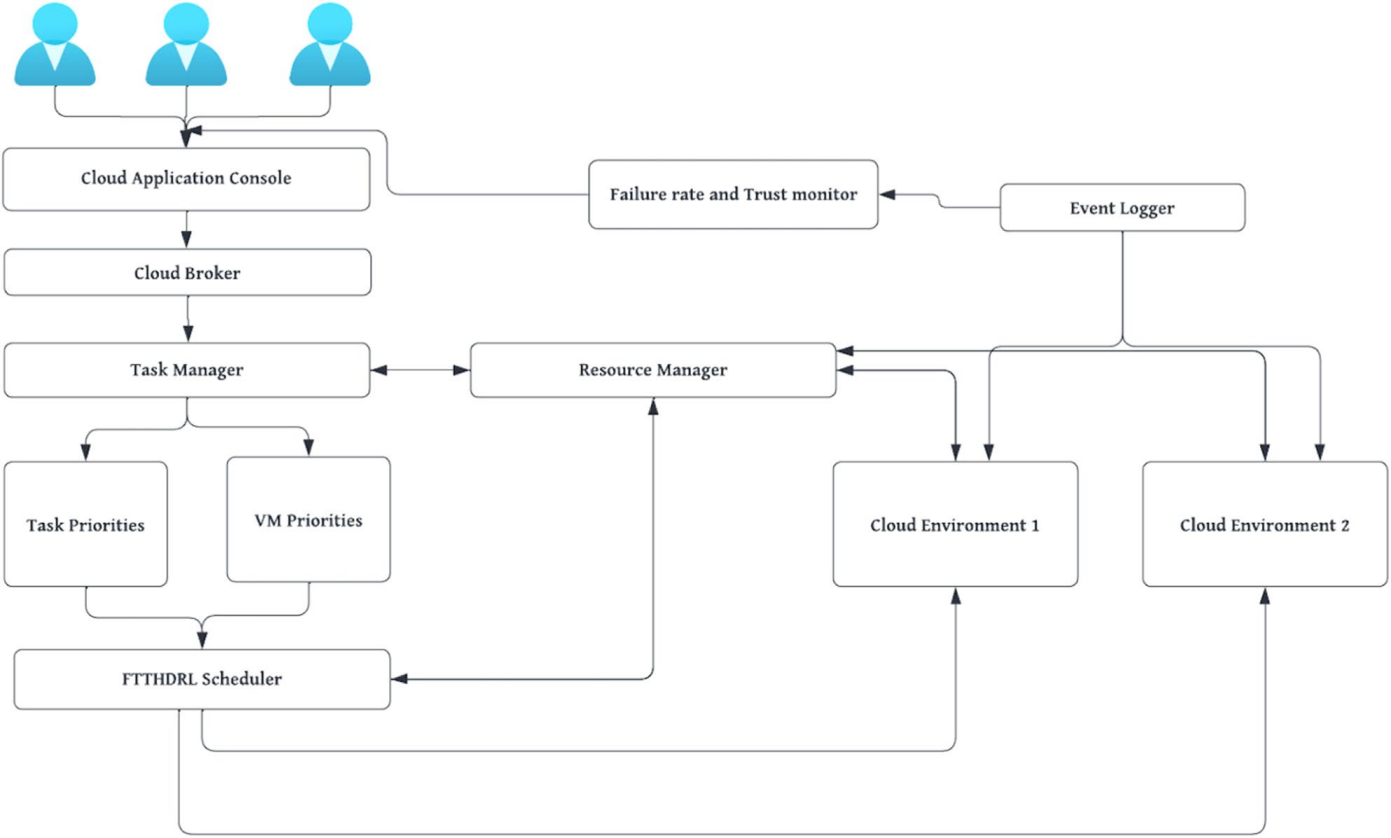
Proposed FTTHDRL system architecture.

In the above encircling mechanism, if the steps of hawk bird is not incremental then hawk encircle prey suddenly by attacking it and represented as Eq. (23) (Fig. 1).

23 $${Z}_{0}=X+{b}_{0}*Lev{y}_{flight}\left(dim\right)$$

24 $$where\,Lev{y}_{flight}\left(X\right)=0.01*\frac{\uptau *\mathrm{n}}{\mid\uprho {\mid }^{\frac{1}{\mathcal{F}}}}$$

25 $$\mathrm{n}={\left(\frac{ \mathrm{n}\left(1+\mathrm{\Finv }\right)*\mathrm{sin}\left(\frac{\mathrm{\pi \Finv }}{2}\right)}{\mathrm{n}\frac{\left(1+\mathrm{\Finv }\right)}{2}*\mathrm{\Finv }*{2}^{\frac{\mathrm{\Finv }-1}{2}}}\right)}^{1/\mathrm{\Finv }}$$

Soft incremental encircling with incremental steps are represented by Eq. (26).

26 $$Z\left( {T + 1} \right) = \left\{ {\begin{array}{*{20}c} {Z_{0} } & {f\left( {Z_{0} } \right) < f\left( {Z\left( T \right)} \right)} \\ X & { f\left( X \right) < f\left( {Z\left( T \right)} \right)} \\ \end{array} } \right.$$

$X,{Z}_{0}$ calculated using Eqs. (22, 23). Hard encircling with incremental steps are represented by Eq. (27).

27 $$Z\left( {T + 1} \right) = \left\{ {\begin{array}{*{20}c} {Z_{0} } & { f\left( {Z_{0} } \right) < f\left( {Z\left( T \right)} \right)} \\ X & {f\left( X \right) < f\left( {Z\left( T \right)} \right)} \\ \end{array} } \right.$$

In above equations ${Z}_{0}, X$ are calculated in Eqs. (22,27) respectively.

28 $${Z}_{0}={Z}^{rab}\left(T\right)-ENER\mid B{Z}^{rab}\left(T\right)-{Z}^{m}\left(T\right)$$

${Z}^{m}\left(T\right)$ is calculated from Eq. (17).

#### Reinforcement Learning based DQN model

This subsection clearly discusses about DQN(Deep Q- network) model which is a reinforcement based learning strategy used as a methodology in our research. This DQN model basically works on Q-learning model. This Q-learning^39^ works based on two tuples in Q-table. They are action space, state space. This is a reinforcement based strategy and therefore it doesn’t need any prior knowledge to generate output. For every iteration of model, this DQN model check for each state with respect to state space and then how it takes action against that corresponding state. A Q-learning function is to be represented using Eq. (29).

29 $$Q\left(stat{e}_{t{a}_{i}}, Ac{t}_{t{a}_{i}}\right)\leftarrow Q\left(stat{e}_{t{a}_{i}}, Ac{t}_{t{a}_{i}}\right)+\Delta *[Re{{w}_{fn}}_{t{a}_{i}}+\omega *\mathrm{max}\left(Q\left(stat{e}_{{\left(ta+1\right)}_{i}},Ac{t}_{ta+1}\right)-Q\left(stat{e}_{t{a}_{i}},Ac{t}_{t{a}_{i}}\right)\right)]$$

For every iteration reinforcement agent looks for Q-learning table keeping in mind that for which state which action to be generated evaluating from Reward function. The outcome of that reward function generates either positive or negative reward based on the input supplied at state space and action space tuples in Q-table but as it is a reinforcement learning based agent it learns from the previous action and improves the generated output accordingly. In Eq. (29). $\Delta$ represents rate of learning for the model, $\omega$ represents discount factor. $stat{e}_{t{a}_{i}}$ represents state space of considered tasks in the model, $Ac{t}_{t{a}_{i}}$ represents action space of considered tasks in model, $stat{e}_{{\left(ta+1\right)}_{i}}$ represents state space of next iteration of considered tasks in the model, $Ac{t}_{ta+1}$ represents action space of next iteration of considered tasks in the model. In this research, while training the agent we used 100 neurons are added as hidden layers in DQN model. Scheduling time for agent to generate a decision is kept as 10 Ms. Replay memory which is used to store states is represented as $rm$. Iterations runs for different number of times and all these values are stored in replay memory and iterations ran as batches. Agent learning time represented as $agen{t}_{time}$.Reward function for this model is represented using Eq. (30).

30 $$Rew_{fn} = \min \left( { Res_{cost} ,ms\left( {ta_{i} } \right),Fail_{rate} } \right), {\text{max}}\left( {trst_{cp} } \right)$$

From the above equation, we evaluate rewards for each of the parameter whether they have improved or not for every iteration we ran in the model. Tables 2 and 3 display the notations and simulation using FTTHDRL system architecture.

**Table 2:** Notations used in FTTHDRL system architecture.

Notation	Meaning
$loa{d}_{{v}_{j}}$	Running tasks on $j$ VMs
$loa{d}_{p{n}_{k}}$	Running tasks on $k$ physical nodes
$tota{l}_{pr{o}_{v{m}_{j}}}$	Total processing capacity of all $j$ VMs
${ta}_{i}^{len}$	Length of $i$ tasks
${ta}_{i}^{pri}$	Priorities of $i$ tasks
${v}_{j}^{pri}$	Priorities of $j$ VMs
$Re{s}_{cost}$	Resource cost in cloud environment
$ms\left(t{a}_{i}\right)$	Makespan of $i$ tasks
$Fai{l}_{rate}$	Fault tolerance or rate of failures of $i$ tasks
$avail\left({v}_{j}\right)$	Availability of $j$ VMs
$su{c}_{rate}\left({v}_{j}\right)$	Success rate of $j$ VMs
$tur{n}_{eff}\left({v}_{j}\right)$	Turn around efficiency of $j$ VMs
$trs{t}_{cp}$	Trust on Cloud provider
$stat{e}_{t{a}_{i}}$	State space of considered $i$ tasks
$Ac{t}_{t{a}_{i}}$	Action space of considered $i$ tasks
$Re{w}_{fn}$	Reward function
$\Delta$	Rate of learning
$\omega$	Discount factor
$rm$	Replay memory

**Table 3:** Simulation settings used in FTTHDRL.

Name	Quantity
No of tasks used in simulation	100–500–1000
Length of tasks used in simulation	100,000
RAM used in VMs	4096 MB
Network bandwidth of VMs	20 Mbps
Processing elements considered for simulation	1800 MIPS
Physical node Memory used for simulation	64 GB
Physical node hard disk capacity for simulation	1 TB
Network Bandwidth of Physical node	200 Mbps
Type of Hypervisor used in simulation	Monolithic
Hypervisor used for simulation	Xen
Operating system of Physical node used in simulation	MAC
Operating System of VM used in simulation	Ubuntu Linux
No. of Datacentres used in multi cloud environment	50

### Proposed FTTHDRL task scheduler in multi cloud environment


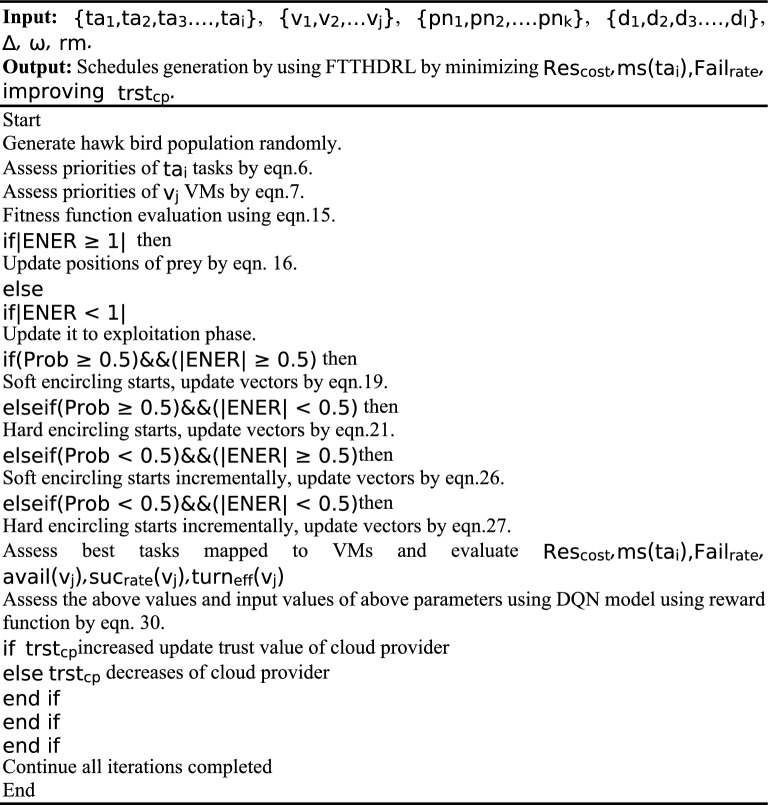


**Figure 2:**
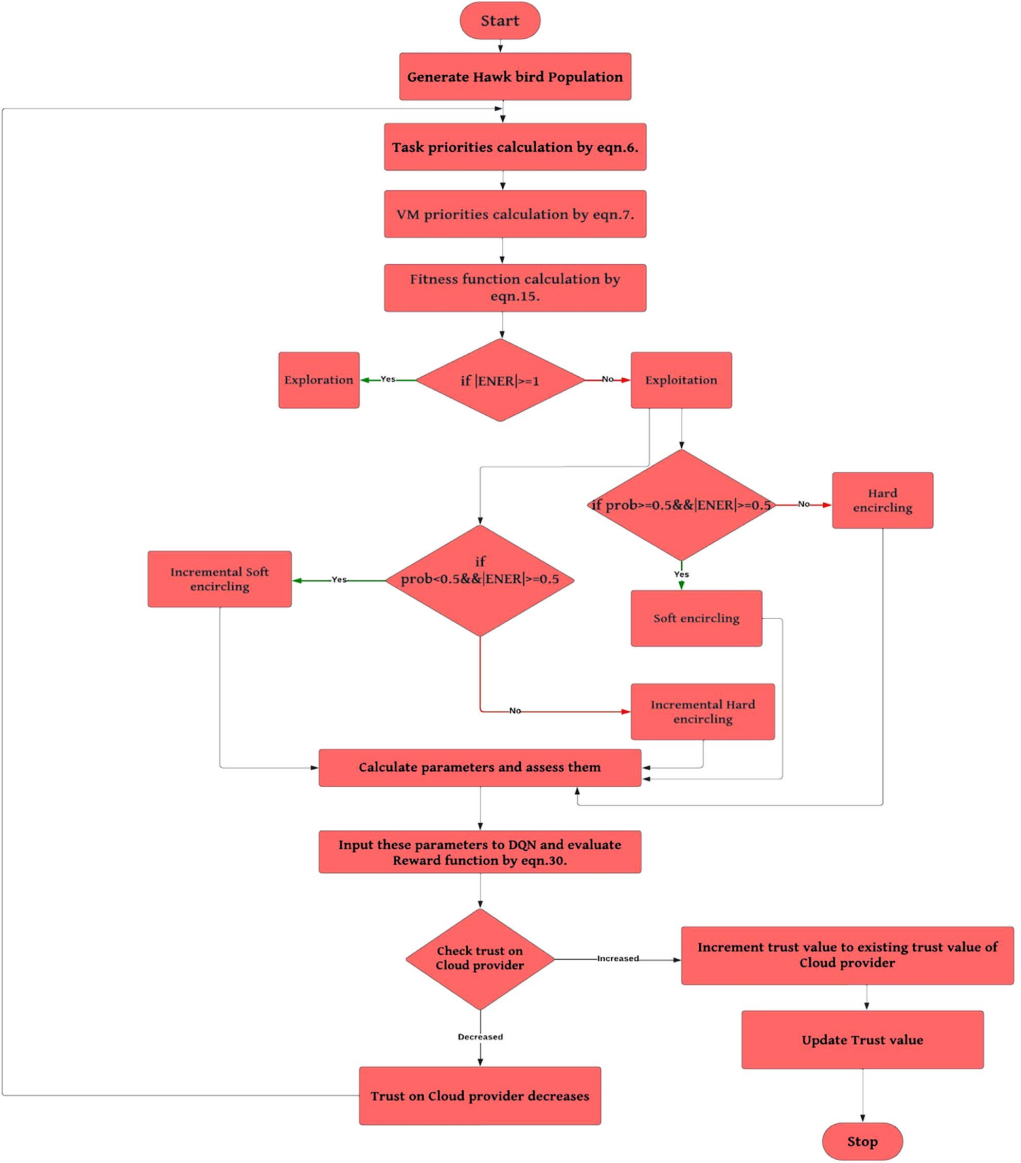
Flow of proposed FTTHDRL scheduler.

The above Fig. 2. shows the flow of our proposed FTTHDRL scheduler which is modelled by hybridization of Harris Hawk algorithm, DQN model which is based on reinforcement learning. Initially, hawk population generated randomly. After that calculation of priorities of tasks, VMs are using by eqns.6, 7. In next step, fitness function calculation using Eq. (15). Algorithm evaluates problem space in two stages by checking a condition $\left|ENER\right|\ge 1$ if it is true then it is in exploitation stage or it is in exploration stage. After this in exploitation if $prob\ge 0.5 \&\& \left|ENER\right|\ge 0.5$ is true then it takes soft encircling on prey otherwise it takes hard encircling. In another step, if $prob\le 0.5 \&\& \left|ENER\right|\ge 0.5$ is true it will go into a stage i.e. Incremental soft encircling otherwise it will be in incremental hard circling. After this step, Assess the parameters and input them to DQN model to generate schedules and evaluate parameters using reward function using Eq. (30) for optimizing parameters. In the next step, assess the SLA based trust parameters. If trust is increased update trust value to existing trust value of cloud provider otherwise trust will be decreased and this process will be continued until all iterations completed. Fig. 1 indicates the physical proposed FTTHDRL system architecture.

## Simulation and results

This section discusses about extensive simulations carried out on Cloudsim tool^40^ for proposed FTTHDRL (Fault tolerant trust based task scheduler using Harris Hawk and deep reinforcement learning). Proposed FTTHDRL simulation performed by giving input parallel worklogs of HPC2N^41^, NASA^42^. Both of these parallel worklogs consists of lengthy, medium, small worklogs. In this research, initially while evaluating and for simulating the algorithm, we fabricated the datasets manually with different statistical distributions which are uniform distributed consists of all tasks are equally distributed. Normal distribution which consists of tasks more medium number of tasks, less number of large, small number of tasks. Left skewed distribution which consists of more large, medium tasks, less number of small tasks. Right skewed presents less number of large, medium, more number of small tasks. Thus, proposed FTTHDRL evaluated in two phases by fabricating workload manually and using real time workload from HPC2N^41^, NASA^42^. In this work we represented Uniform, Normal, Left, right distributions as U01, N02, L03, R04, H05, NA06 respectively. The below Section “Configuration settings used in FTTHDRL for simulation” presents configuration settings of simulation which are used in simulation.

### Configuration settings used in FTTHDRL for simulation

This Section “Configuration settings used in FTTHDRL for simulation” discusses simulation settings used in proposed FTTHDRL and we captured standard simulation settings from ^43^. We have installed Cloudsim tool^40^ in our environment. It was installed in MAC operating system, 64 GB RAM, 8-core CPU with M1 chip resided in it.

### Evaluation of makespan for FTTHDRL

**Figure 3:**
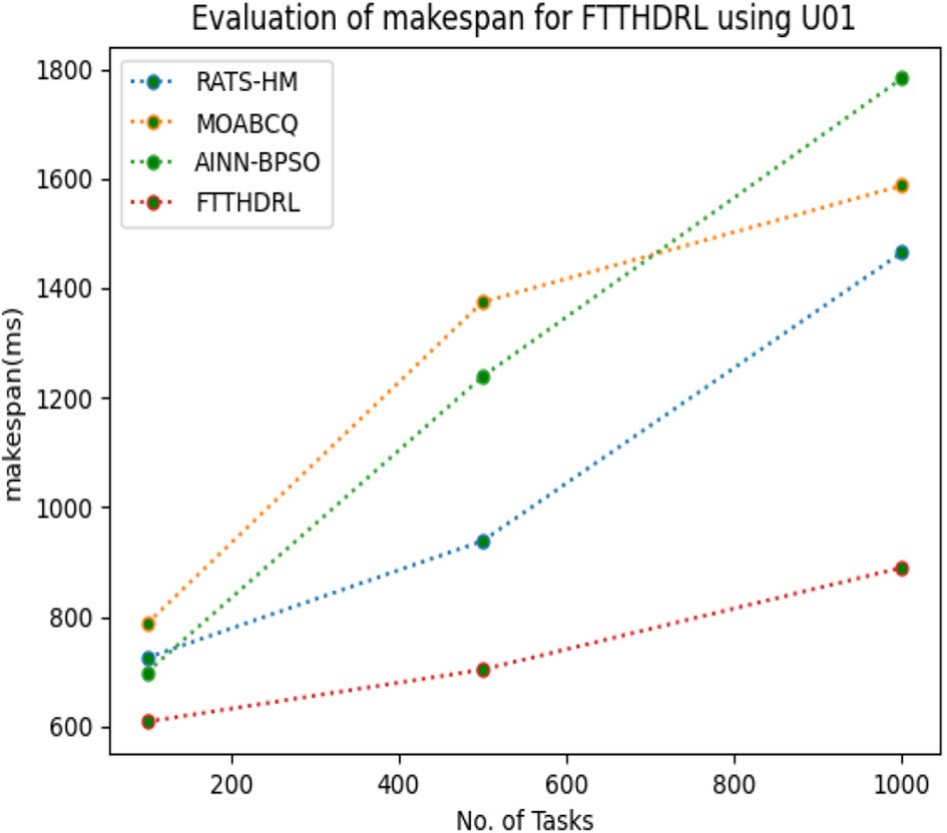
Makespan evaluation by U01.

**Figure 4:**
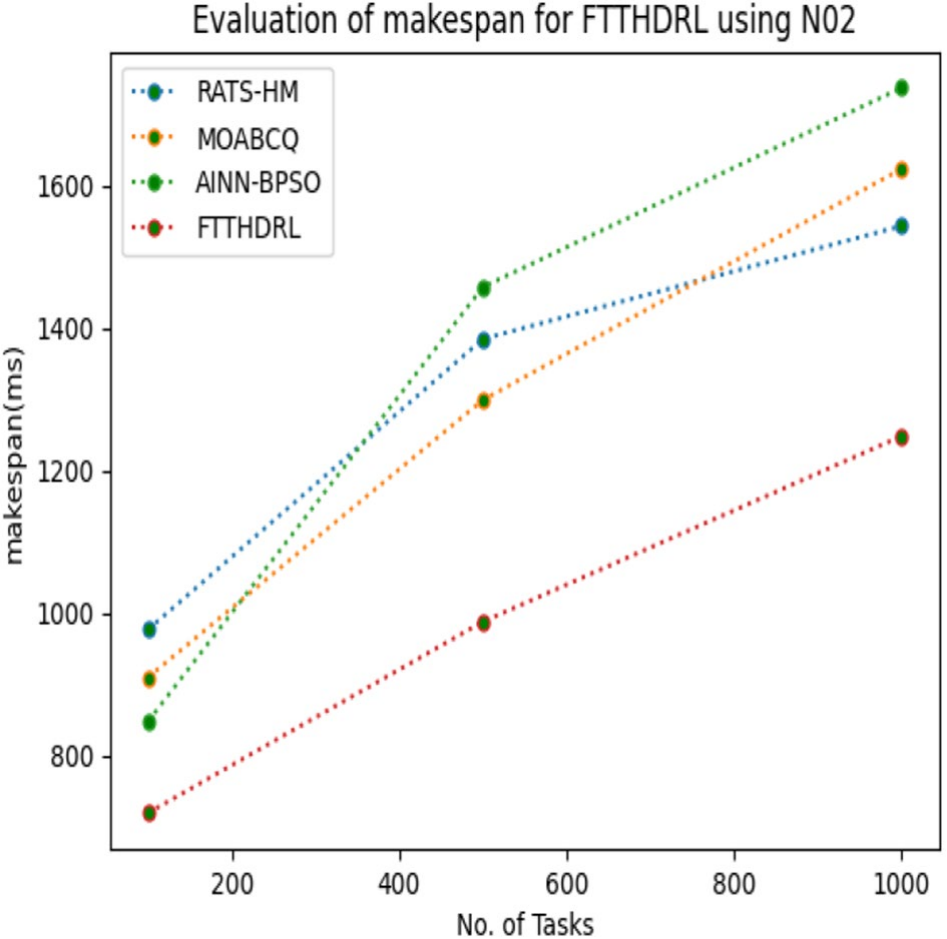
Makespan evaluation by N02.

**Figure 5:**
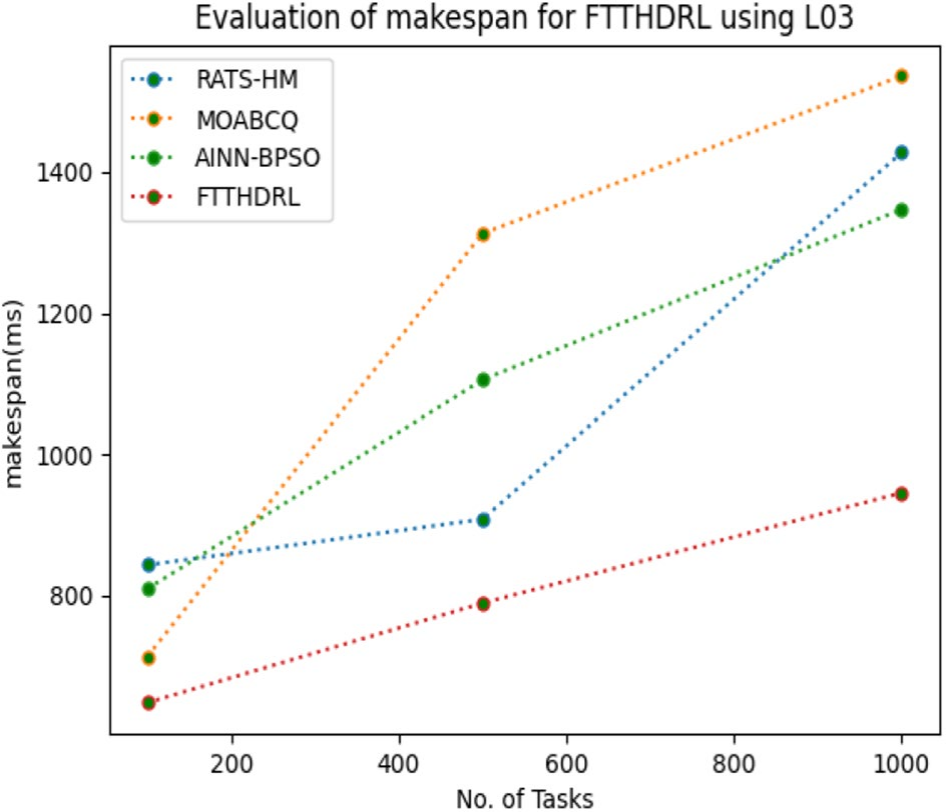
Makespan evaluation by L03.

**Figure 6:**
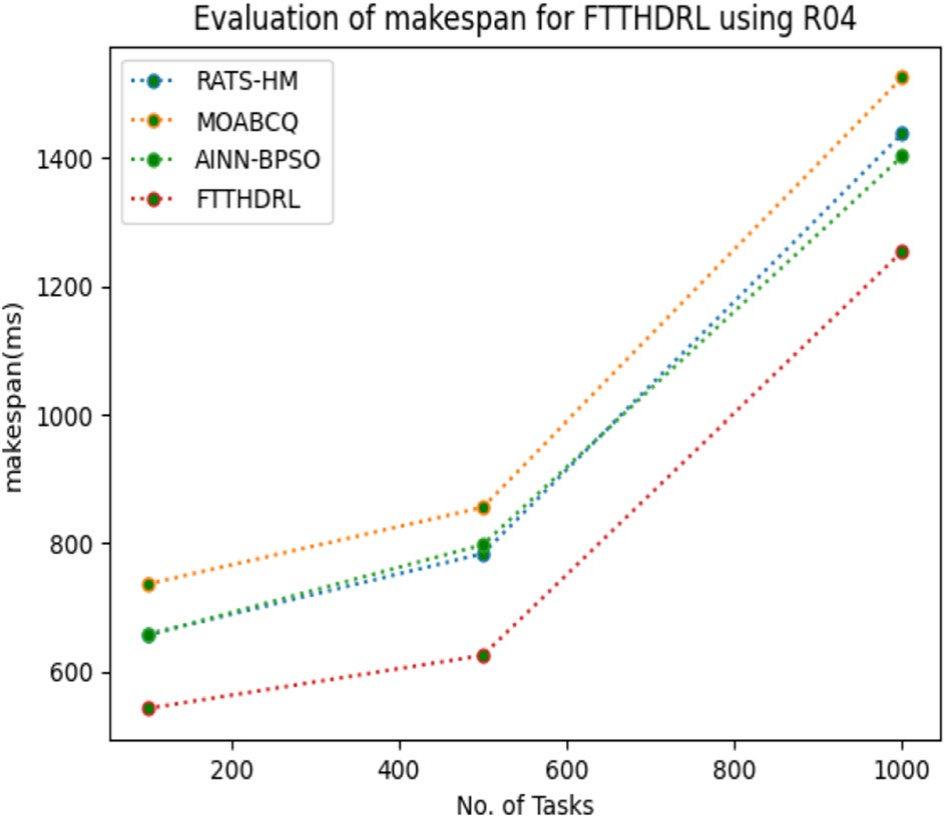
Makespan evaluation by R04.

**Figure 7:**
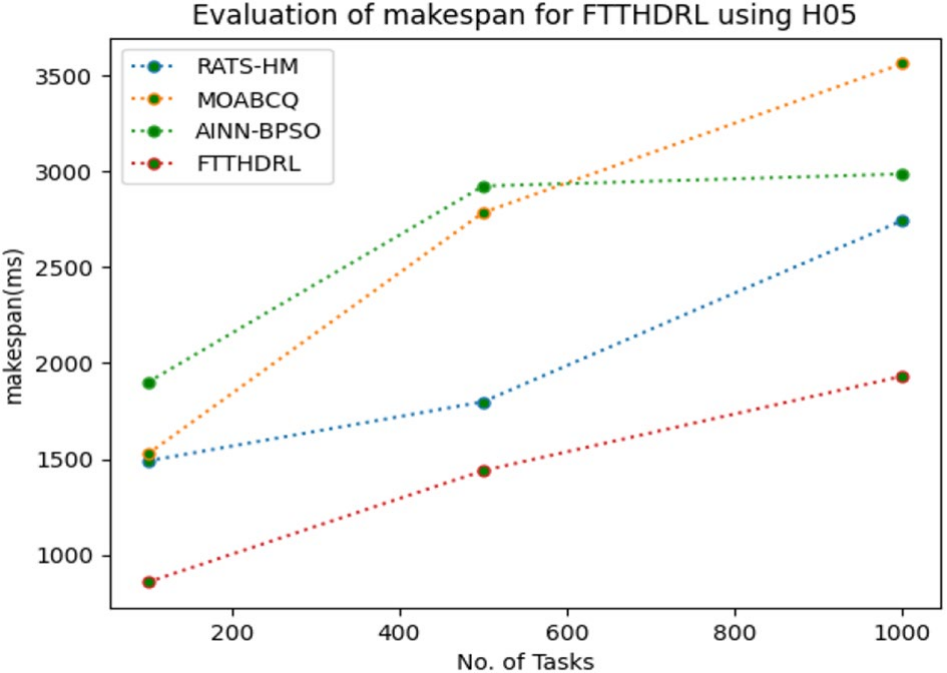
Makespan evaluation by H05.

**Figure 8:**
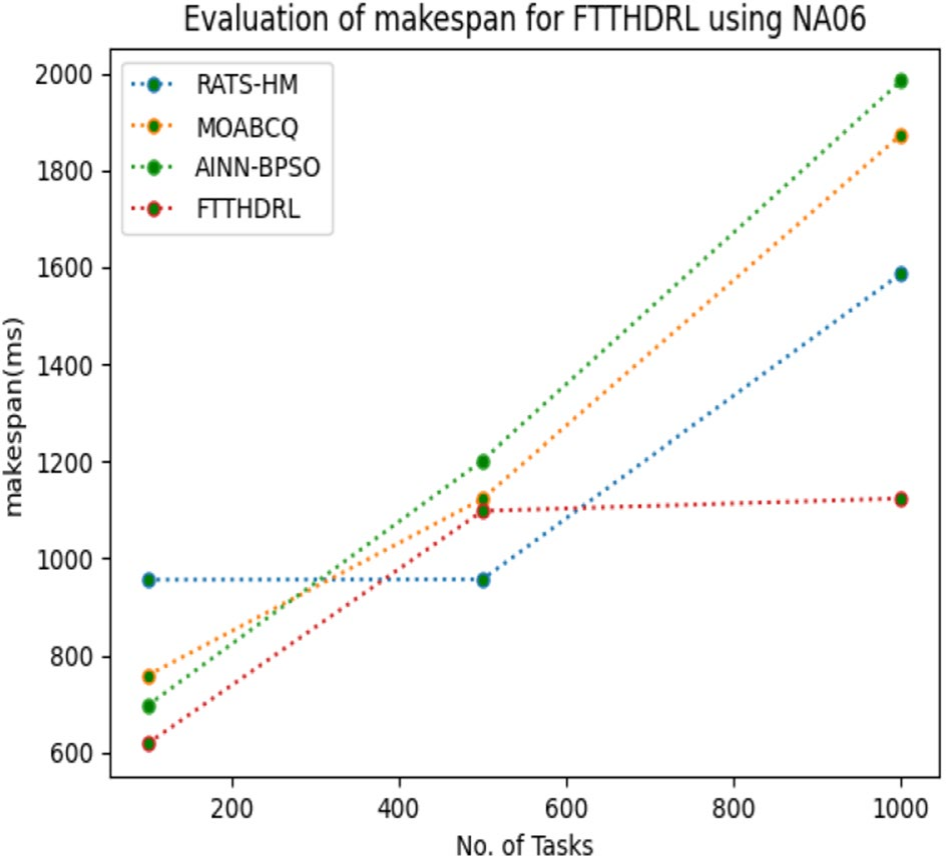
Makespan evaluation by NA06.

In this Section “Evaluation of makespan for FTTHDRL”, we carefully evaluated makespan of our proposed FTTHDRL by giving input workload from various fabricated workloads from U01, N02, L03, R04 and real time parallel worklogs from H05, NA06. Proposed FTTHDRL compared over existing RATS-HM, MOABCQ, AINN-BPSO approaches. Table 4 represents generated makespan of 100, 500, 1000 tasks. Figures 3, 4, 5, 6, 7, 8 represents makespan of U01, N02, L03, R04, H05, NA06 respectively. After observing results generated from makespan FTTHDRL improves makespan greatly over state of art algorithms.

**Table 4:** Evaluation of makespan for FTTHDRL.

No. of Tasks	RATS-HM	MOABCQ	AINN-BPSO	FTTHDRL
100(U01)	724.87	787.17	698.65	608.19
500(U01)	938.29	1374.54	1238.24	703.17
1000(U01)	1465.15	1587.21	1783.14	889.56
100(N02)	976.29	909.15	847.24	718.84
500(N02)	1384.36	1298.78	1457.39	987.45
1000(N02)	1543.15	1623.51	1737.11	1248.21
100(L03)	843.18	712.99	809.26	647.75
500(L03)	908.14	1312.78	1105.17	789.11
1000(L03)	1427.53	1536.12	1347.02	945.57
100(R04)	658.37	736.77	657.11	543.21
500(R04)	784.22	856.16	798.17	625.87
1000(R04)	1437.42	1524.77	1402.11	1254.11
100(H05)	1489.36	1524.87	1899.28	856.18
500(H05)	1798.18	2786.32	2924.58	1438.76
1000(H05)	2745.12	3564.12	2987.67	1932.17
100(NA06)	956.37	758.93	696.74	618.17
500(NA06)	957.12	1123.12	1202.13	1098.15
1000(NA06)	1587.09	1873.29	1984.21	1124.11

### Evaluation of rate of failures for FTTHDRL

**Figure 9:**
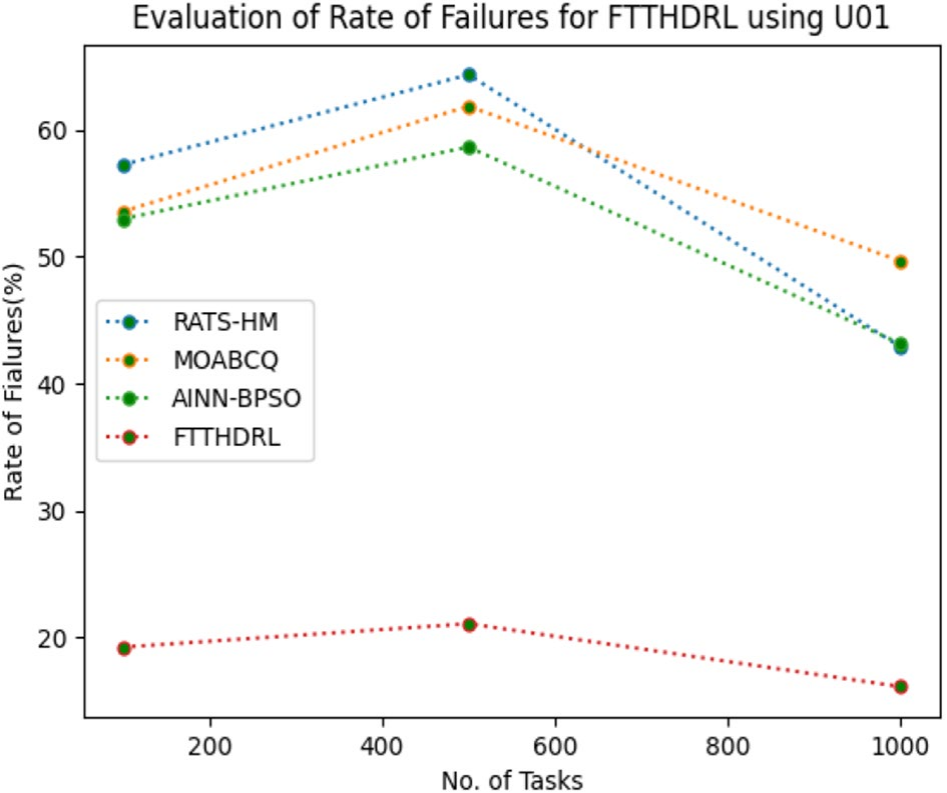
Rate of Failures using U01.

**Figure 10:**
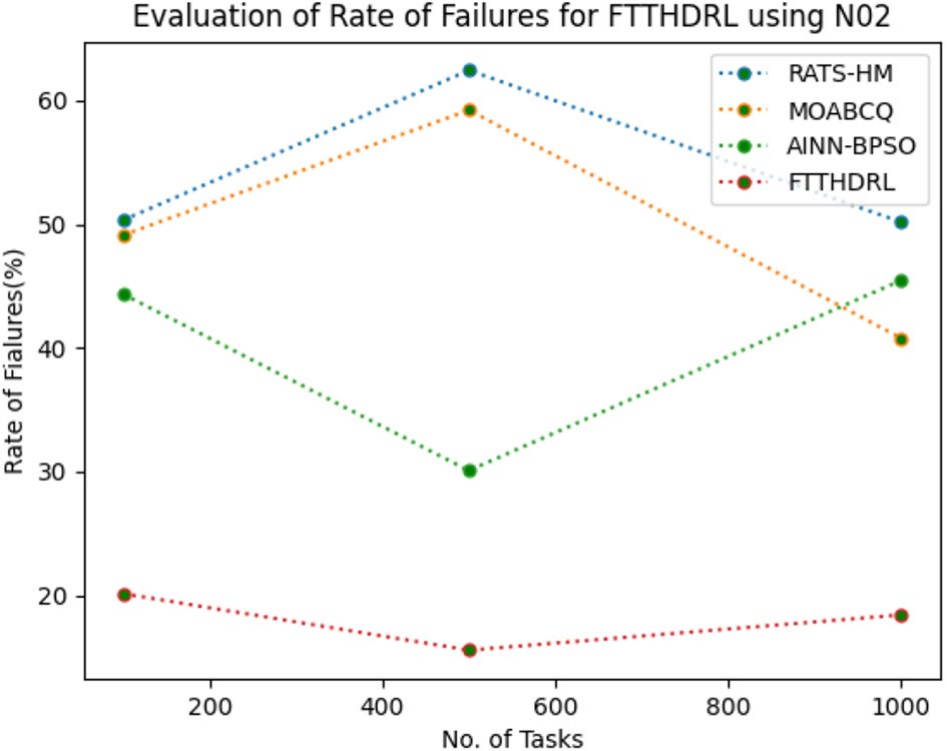
Rate of Failures using N02.

**Figure 11:**
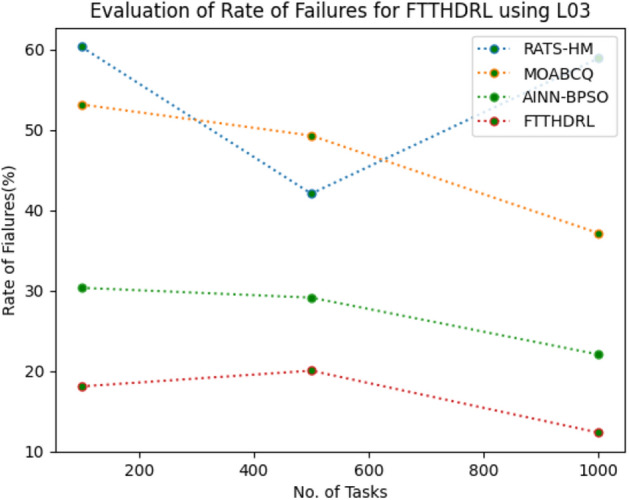
Rate of Failures using L03.

**Figure 12:**
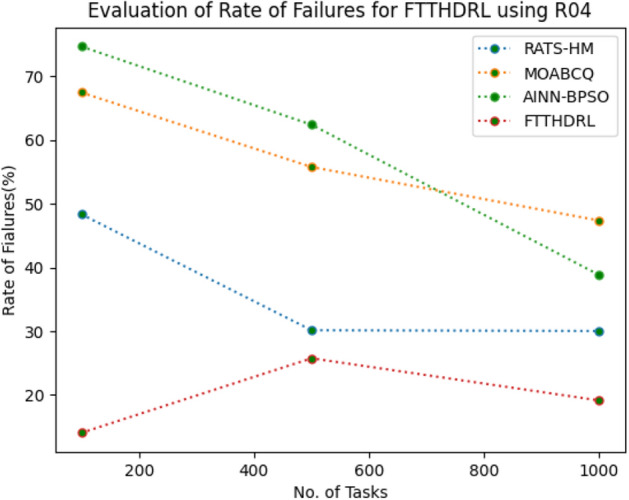
Rate of Failures using R04.

**Figure 13:**
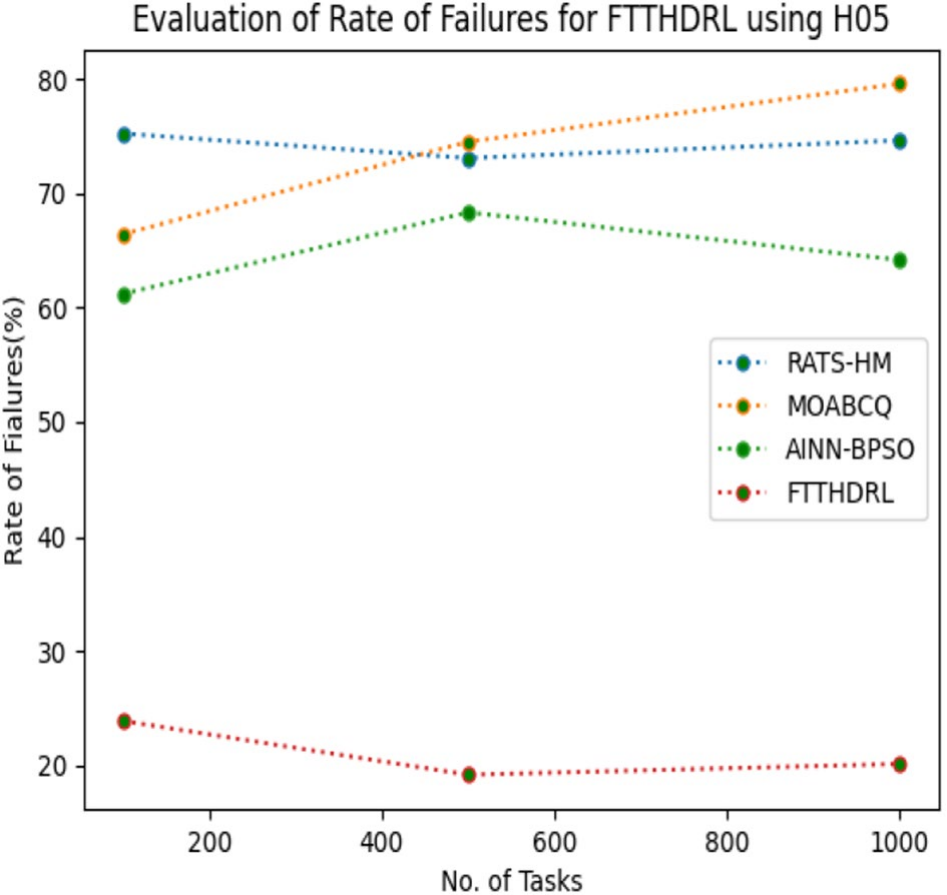
Rate of Failures using H05.

**Figure 14:**
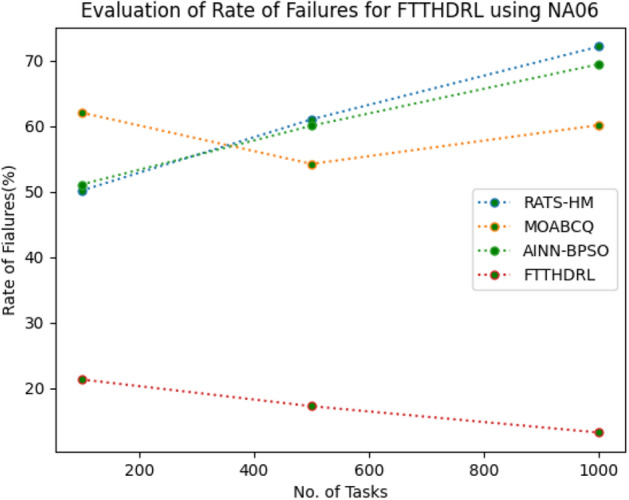
Rate of Failures using NA06.

In this Section “Evaluation of rate of failures for FTTHDRL”, we evaluated Rate of failures of our proposed FTTHDRL by giving input workload from various fabricated workloads from U01, N02, L03, R04 and real time parallel worklogs from H05, NA06. Proposed FTTHDRL compared over existing RATS-HM, MOABCQ, AINN-BPSO approaches. Table 5 represents Rate of failures for 100, 500, 1000 tasks. Figures 9, 10, 11, 12, 13, 14 represents rate of failures of U01, N02, L03, R04, H05, NA06 respectively. After observing results generated rate of failures for FTTHDRL minimizes rate of failures greatly over state of art algorithms.

**Table 5:** Evaluation of rate of failures for FTTHDRL.

Tasks	RATS-HM	MOABCQ	AINN-BPSO	FTTHDRL
100(U01)	57.25	53.57	52.98	19.24
500(U01)	64.36	61.87	58.66	21.09
1000(U01)	42.87	49.65	43.12	16.12
100(N02)	50.32	49.11	44.35	20.14
500(N02)	62.43	59.21	30.08	15.57
1000(N02)	50.16	40.78	45.47	18.42
100(L03)	60.33	53.16	30.37	18.11
500(L03)	42.07	49.29	29.15	20.07
1000(L03)	58.91	37.15	22.08	12.36
100(R04)	48.36	67.46	74.68	14.06
500(R04)	30.16	55.77	62.41	25.76
1000(R04)	30.04	47.37	38.82	19.14
100(H05)	75.26	66.43	61.21	23.86
500(H05)	73.11	74.54	68.35	19.14
1000(H05)	74.66	79.63	64.19	20.09
100(NA06)	50.17	62.06	51.10	21.37
500(NA06)	61.03	54.24	60.06	17.29
1000(NA06)	72.15	60.17	69.46	13.27

### Evaluation of availability for FTTHDRL

**Figure 15:**
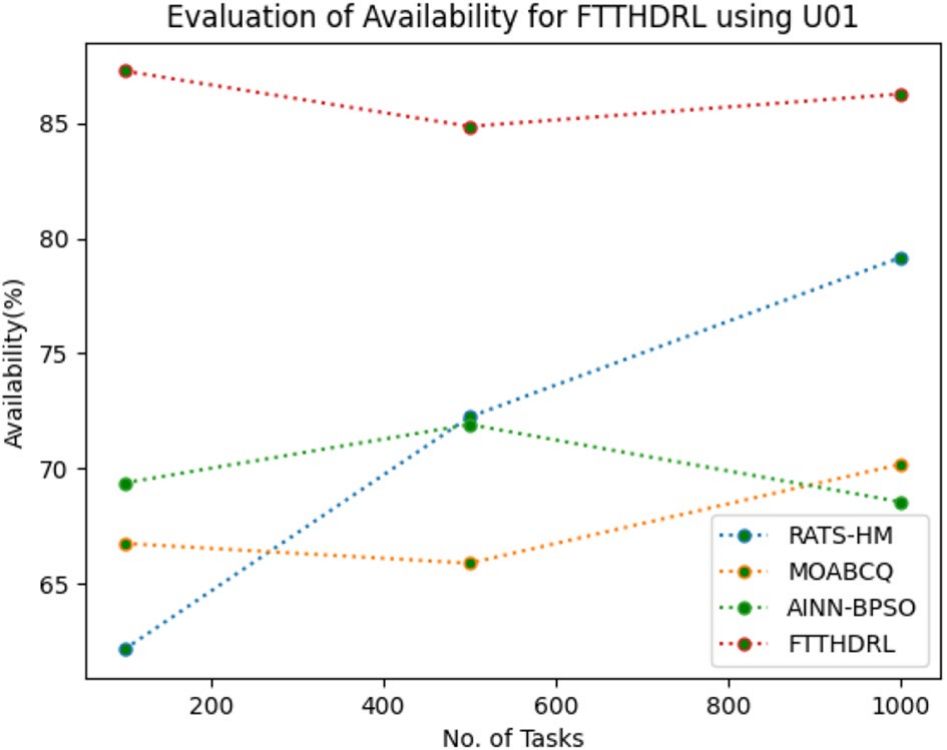
Availability of VMs using U01.

**Figure 16:**
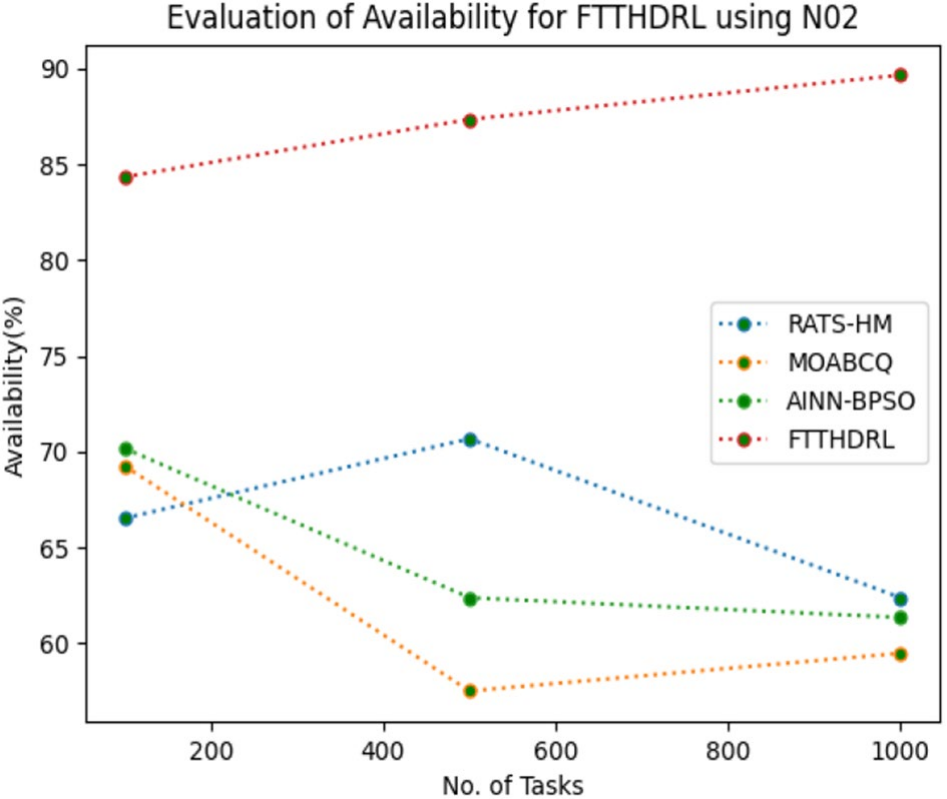
Availability of VMs using N02.

**Figure 17:**
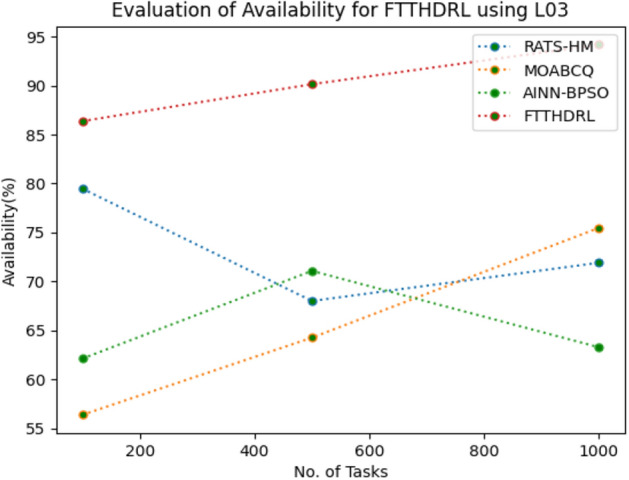
Availability of VMs using L03.

**Figure 18:**
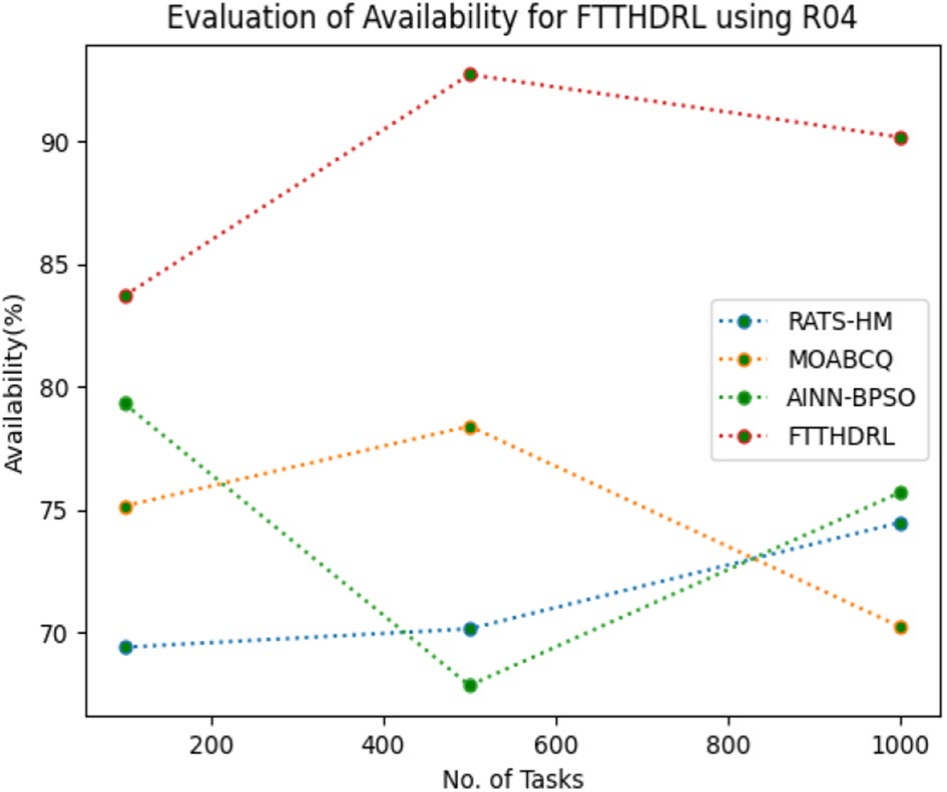
Availability of VMs using R04.

**Figure 19:**
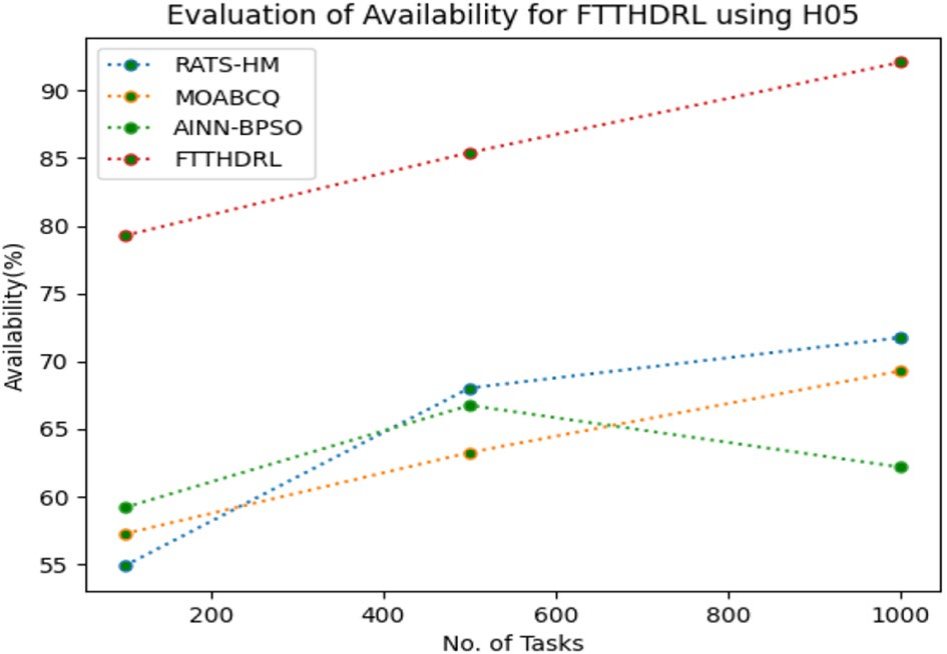
Availability of VMs using H05.

**Figure 20:**
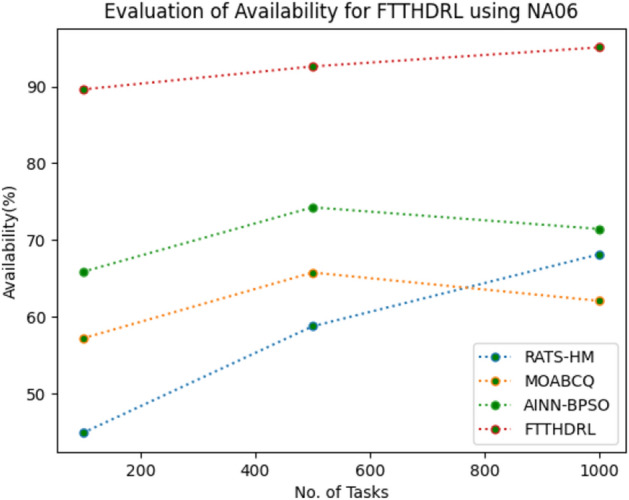
Availability of VMs using NA06.

In this Section “Evaluation of availability for FTTHDRL”, we evaluated Availability of VMs of our proposed FTTHDRL by giving input workload from various fabricated workloads from U01, N02, L03, R04 and real time parallel worklogs from H05, NA06. Proposed FTTHDRL compared over existing RATS-HM, MOABCQ, AINN-BPSO approaches. Table 6 represents availability of VMs for 100, 500, 1000 tasks. Figures 15, 16, 17, 18, 19, 20 represents availability of VMs for U01, N02, L03, R04, H05, NA06 respectively. After observing results generated availability for FTTHDRL improves availability greatly over state of art algorithms.

**Table 6:** Evaluation of availability of VMs for FTTHDRL.

Tasks	RATS-HM	MOABCQ	AINN-BPSO	FTTHDRL
100(U01)	62.13	66.74	69.37	87.28
500(U01)	72.24	65.88	71.91	84.87
1000(U01)	79.18	70.19	68.54	86.28
100(N02)	66.53	69.27	70.18	84.35
500(N02)	70.68	57.51	62.38	87.36
1000(N02)	62.38	59.48	61.35	89.67
100(L03)	79.48	56.38	62.12	86.37
500(L03)	68.01	64.26	71.08	90.14
1000(L03)	71.91	75.48	63.28	94.18
100(R04)	69.42	75.17	79.35	83.72
500(R04)	70.17	78.43	67.86	92.74
1000(R04)	74.51	70.24	75.74	90.18
100(H05)	54.87	57.25	59.19	79.27
500(H05)	68.04	63.27	66.76	85.42
1000(H05)	71.77	69.31	62.18	92.10
100(NA06)	44.86	57.19	65.86	89.63
500(NA06)	58.76	65.77	74.27	92.63
1000(NA06)	68.17	62.07	71.44	95.12

### Evaluation of success rate for FTTHDRL

**Figure 21:**
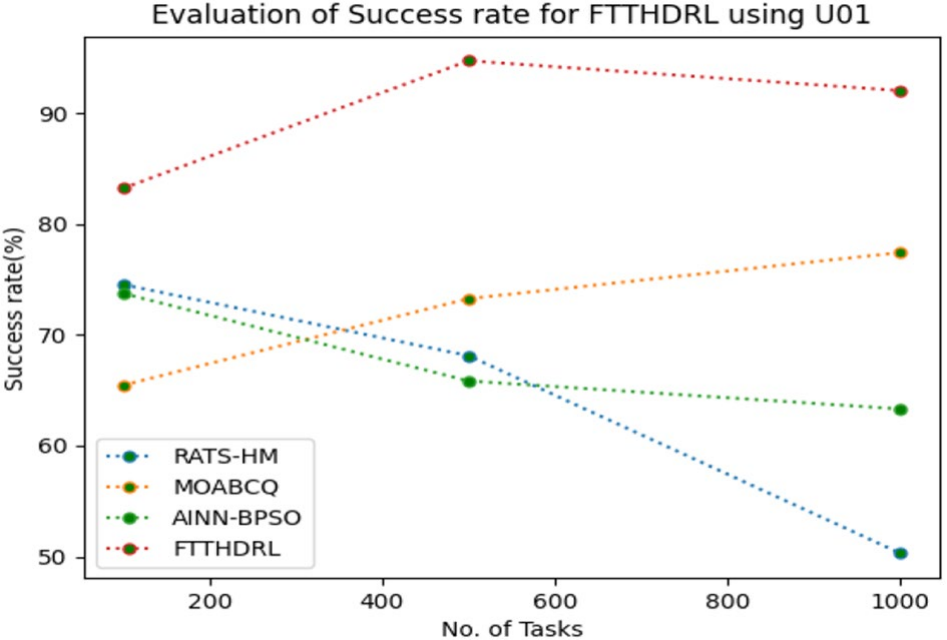
Success rate of VMs using U01.

**Figure 22:**
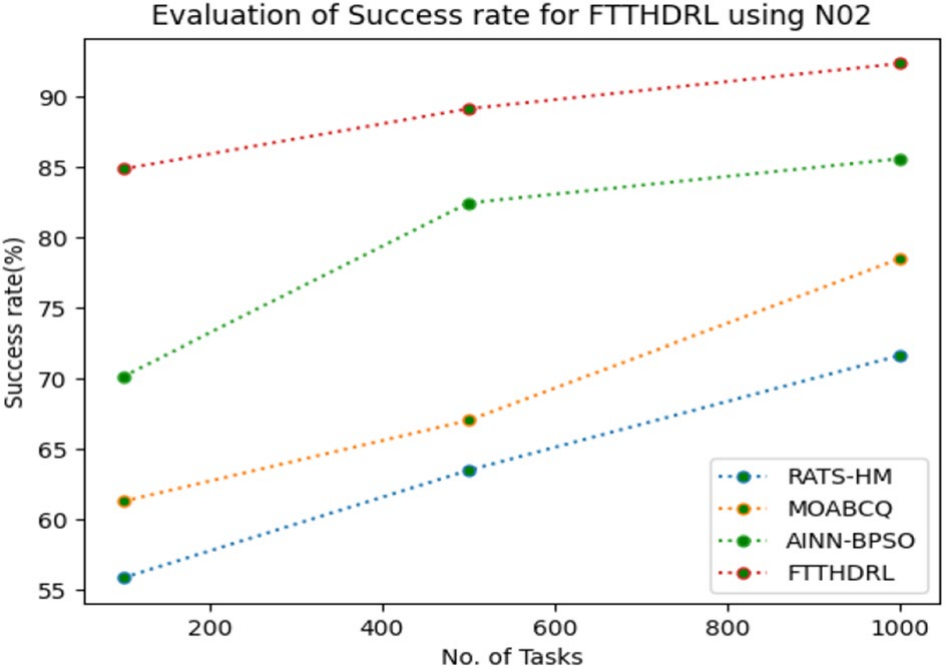
Success rate of VMs using N02.

**Figure 23:**
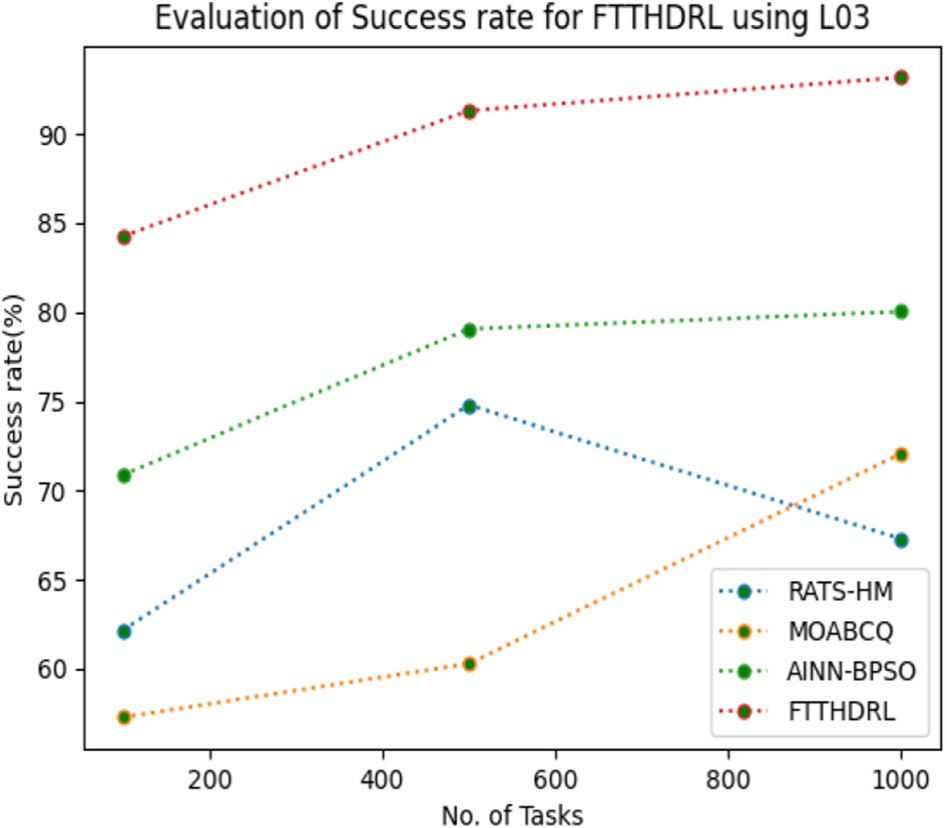
Success rate of VMs using L03.

**Figure 24:**
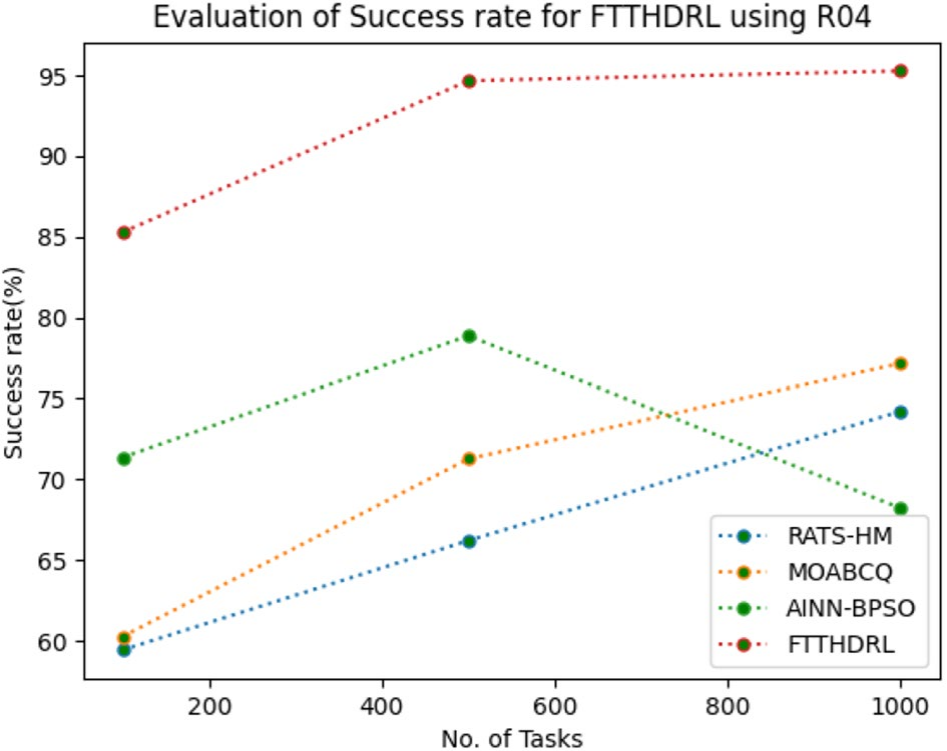
Success rate of VMs using R04.

**Figure 25:**
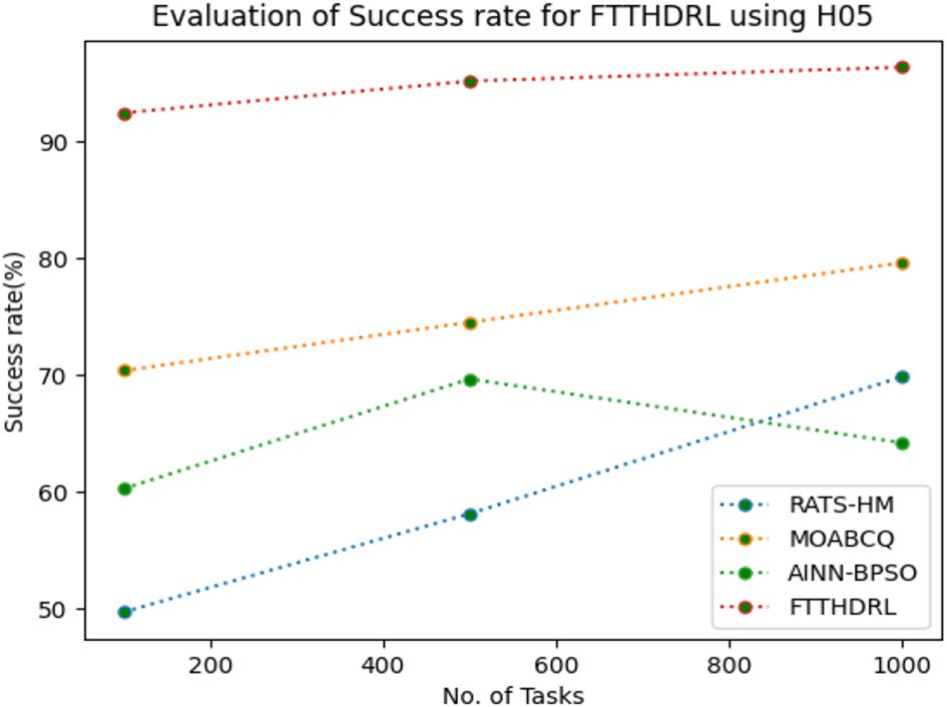
Success rate of VMs using H05.

**Figure 26:**
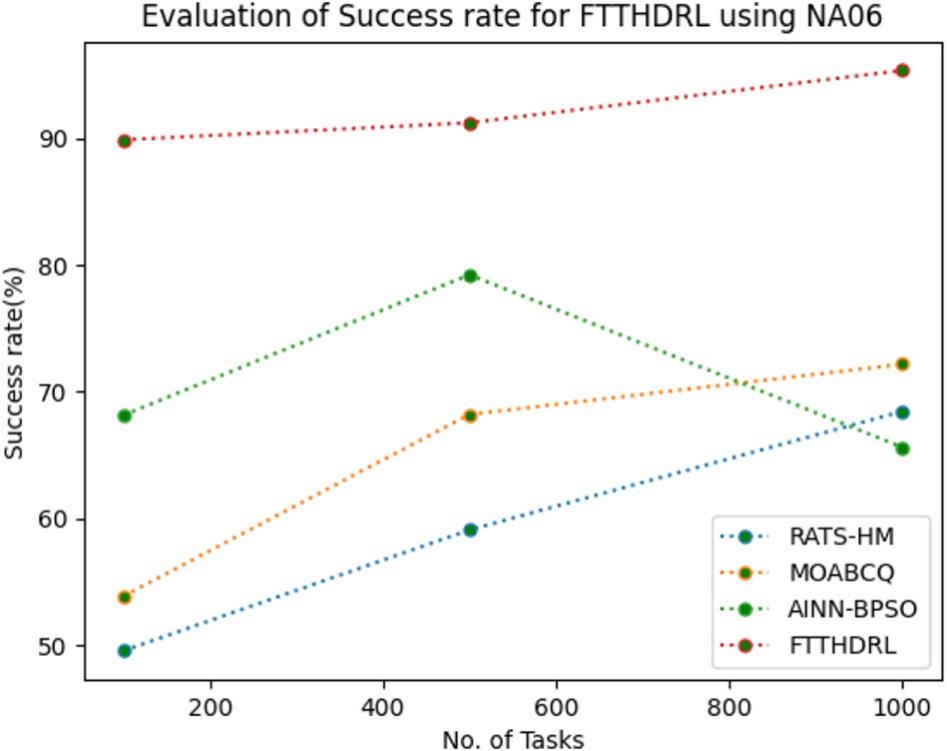
Success rate of VMs using NA06.

In this Section “Evaluation of success rate for FTTHDRL”, we evaluated Success rate of VMs of our proposed FTTHDRL by giving input workload from various fabricated workloads from U01, N02, L03, R04 and real time parallel worklogs from H05, NA06. Proposed FTTHDRL compared over existing RATS-HM, MOABCQ, AINN-BPSO approaches. Table 7 represents Success rate of VMs for 100, 500, 1000 tasks. Figures 21, 22, 23, 24, 25, 26 represents success rate of VMs for U01, N02, L03, R04, H05, NA06 respectively. After observing results generated Success rate for FTTHDRL improves availability greatly over state of art algorithms.

**Table 7:** Evaluation of success rate of VMs for FTTHDRL.

Tasks	RATS-HM	MOABCQ	AINN-BPSO	FTTHDRL
100(U01)	74.56	65.46	73.76	83.24
500(U01)	68.13	73.28	65.85	94.76
1000(U01)	50.28	77.45	63.32	92.06
100(N02)	55.84	61.28	70.14	84.87
500(N02)	63.49	67.04	82.46	89.16
1000(N02)	71.67	78.52	85.61	92.38
100(L03)	62.15	57.26	70.88	84.26
500(L03)	74.78	60.27	79.05	91.32
1000(L03)	67.28	72.09	80.03	93.19
100(R04)	59.43	60.26	71.36	85.28
500(R04)	66.21	71.27	78.87	94.67
1000(R04)	74.22	77.17	68.21	95.28
100(H05)	49.67	70.37	60.25	92.46
500(H05)	58.11	74.52	69.66	95.19
1000(H05)	69.87	79.63	64.18	96.38
100(NA06)	49.57	53.87	68.15	89.87
500(NA06)	59.11	68.22	79.24	91.22
1000(NA06)	68.47	72.18	65.62	95.35

### Evaluation of Turnaround efficiency for FTTHDRL

**Figure 27:**
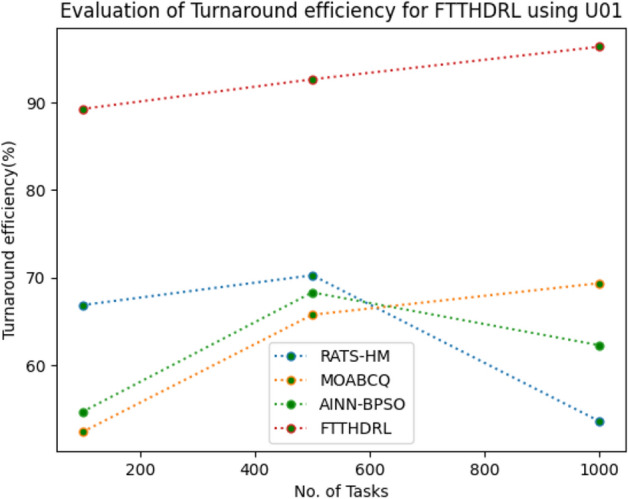
Turnaround efficiency of VMs using U01.

**Figure 28:**
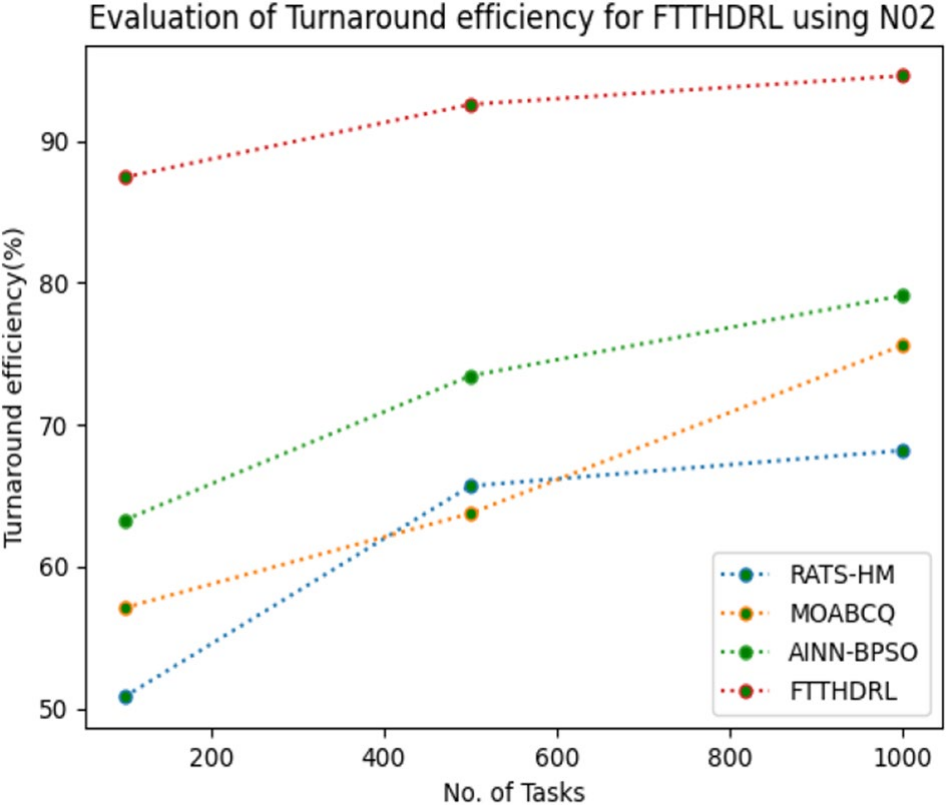
Turnaround efficiency of VMs using N02.

**Figure 29:**
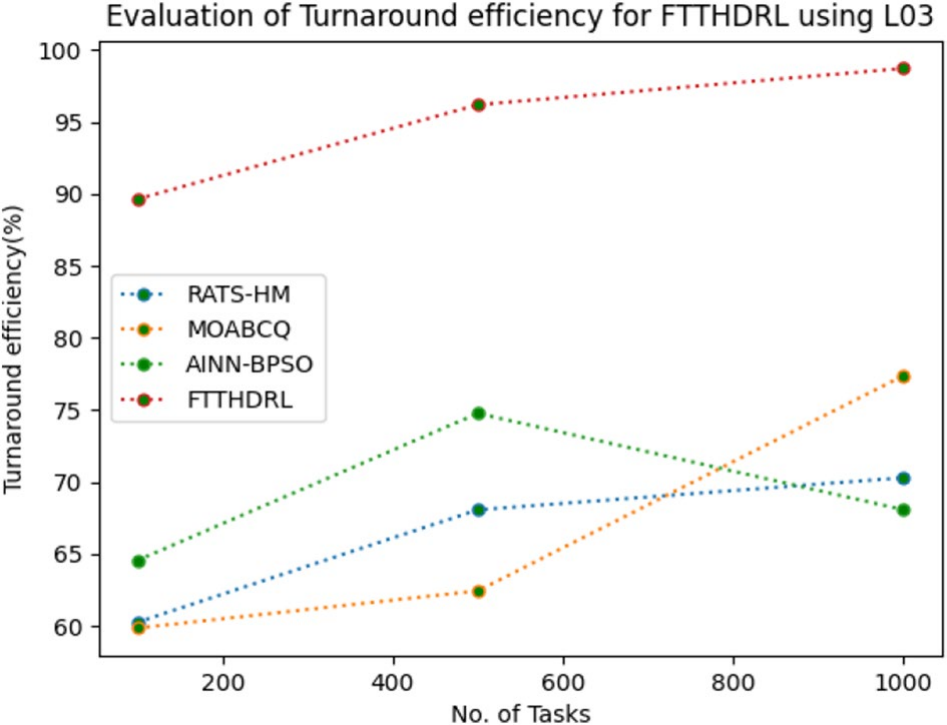
Turnaround efficiency of VMs using L03.

**Figure 30:**
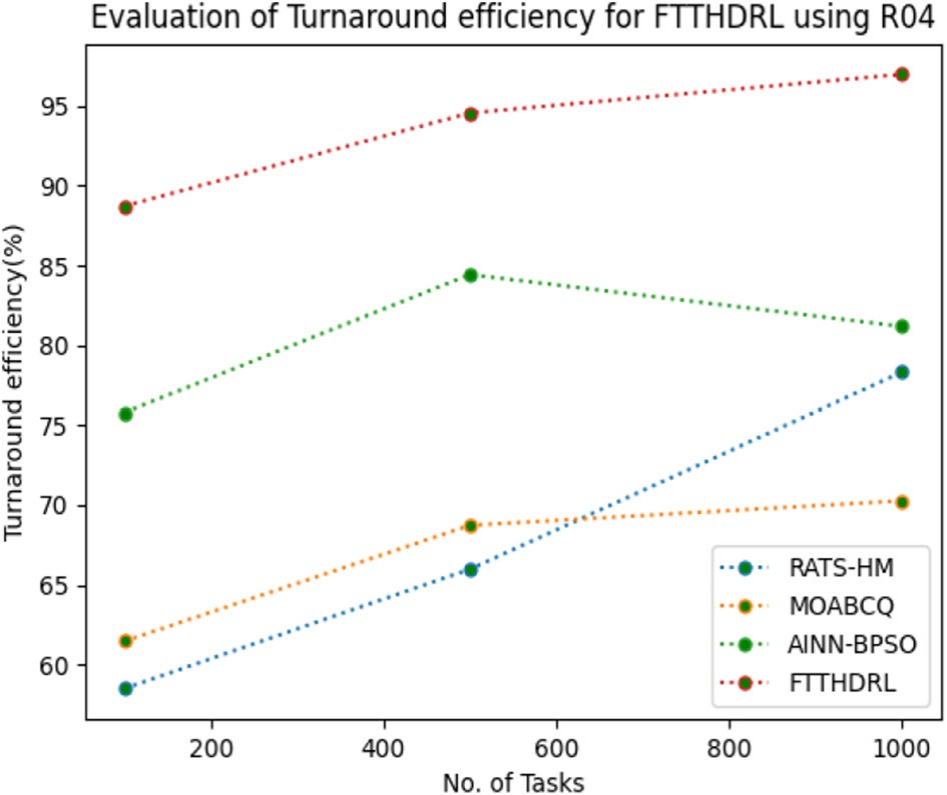
Turnaround efficiency of VMs using R04.

**Figure 31:**
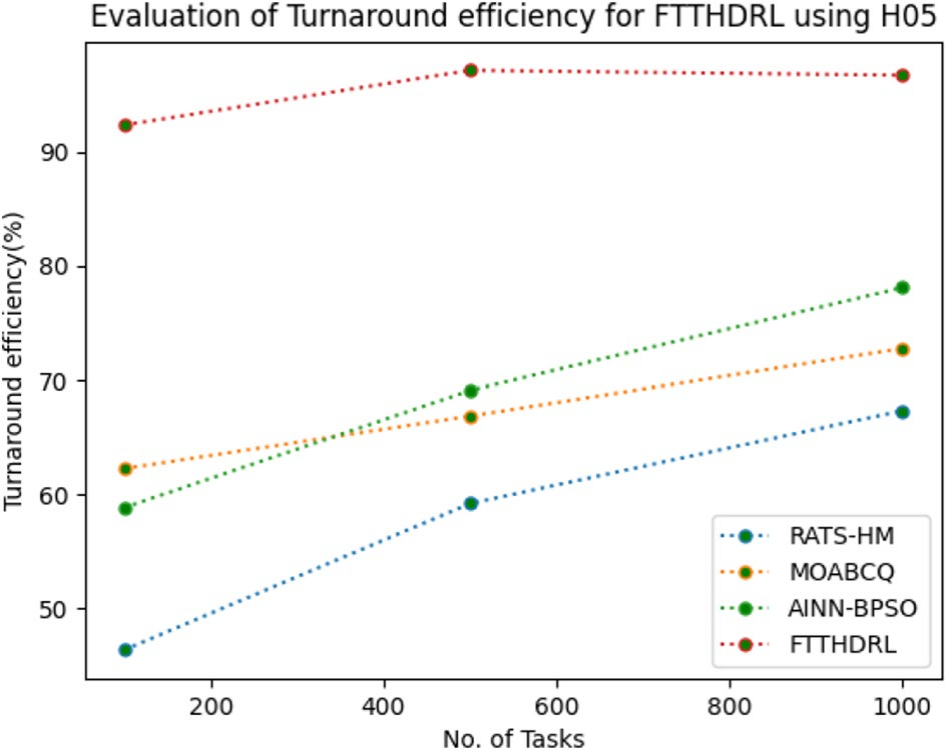
Turnaround efficiency of VMs using H05.

**Figure 32:**
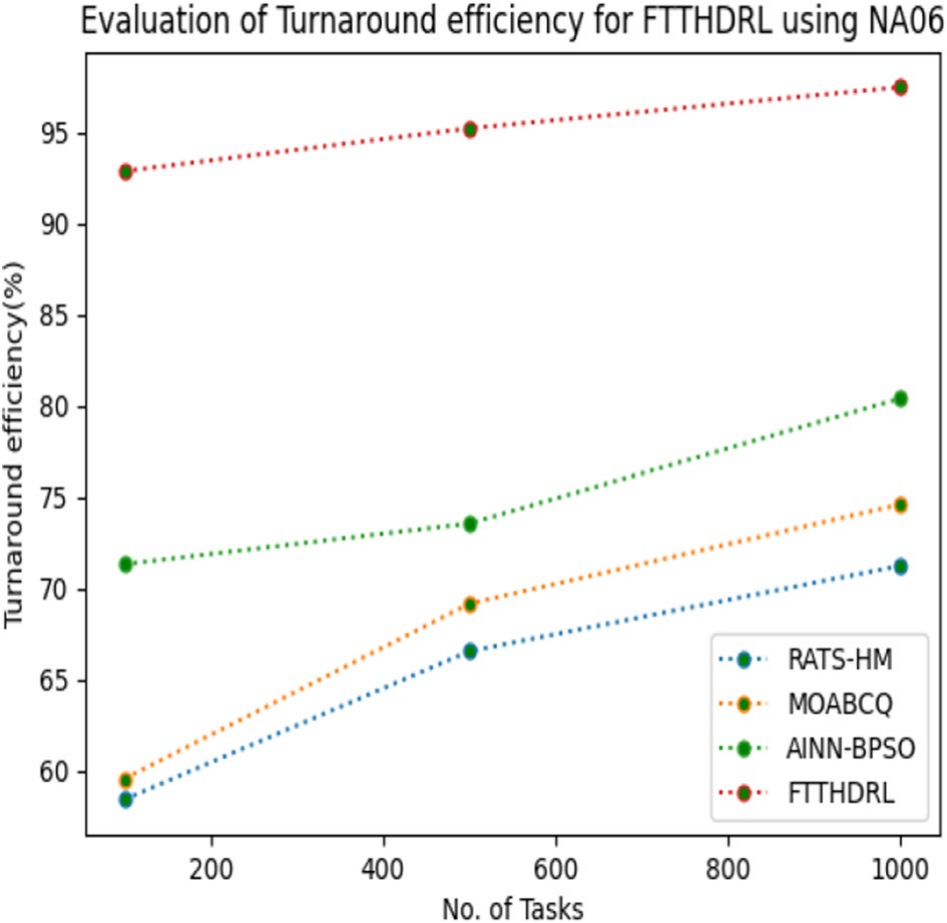
Turnaround efficiency of VMs using NA06.

In this Section “Evaluation of Turnaround efficiency for FTTHDRL”, we evaluated Turnaround efficiency of VMs of our proposed FTTHDRL by giving input workload from various fabricated workloads from U01, N02, L03, R04 and real time parallel worklogs from H05, NA06. Proposed FTTHDRL compared over existing RATS-HM, MOABCQ, AINN-BPSO approaches. Table 8 represents Turnaround efficiency for 100, 500, 1000 tasks. Figures 27, 28, 29, 30, 31, 32 represents Turnaround efficiency for U01, N02, L03, R04, H05, NA06 respectively. After observing results generated Turnaround efficiency for FTTHDRL improves Turnaround efficiency greatly over state of art algorithms.

**Table 8:** Evaluation of Turnaround efficiency of VMs for FTTHDRL.

Tasks	RATS-HM	MOABCQ	AINN-BPSO	FTTHDRL
100(U01)	66.87	52.36	54.67	89.28
500(U01)	70.28	65.76	68.32	92.67
1000(U01)	53.58	69.38	62.28	96.39
100(N02)	50.82	57.10	63.27	87.42
500(N02)	65.68	63.75	73.44	92.56
1000(N02)	68.19	75.61	79.11	94.58
100(L03)	60.26	59.86	64.56	89.63
500(L03)	68.08	62.45	74.78	96.19
1000(L03)	70.31	77.41	68.08	98.72
100(R04)	58.49	61.46	75.78	88.72
500(R04)	65.98	68.73	84.43	94.56
1000(R04)	78.32	70.27	81.17	96.99
100(H05)	46.36	62.27	58.85	92.37
500(H05)	59.21	66.88	69.12	97.18
1000(H05)	67.36	72.78	78.12	96.75
100(NA06)	58.43	59.57	71.34	92.87
500(NA06)	66.56	69.17	73.57	95.22
1000(NA06)	71.26	74.62	80.41	97.48

### Evaluation of resource cost for FTTHDRL

**Figure 33:**
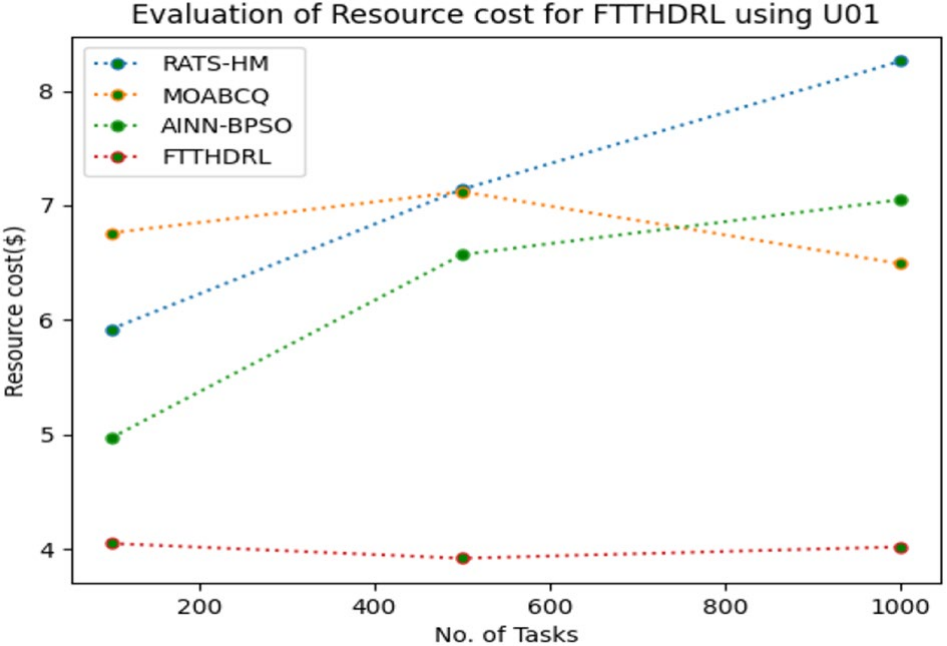
Resource cost using U01.

**Figure 34:**
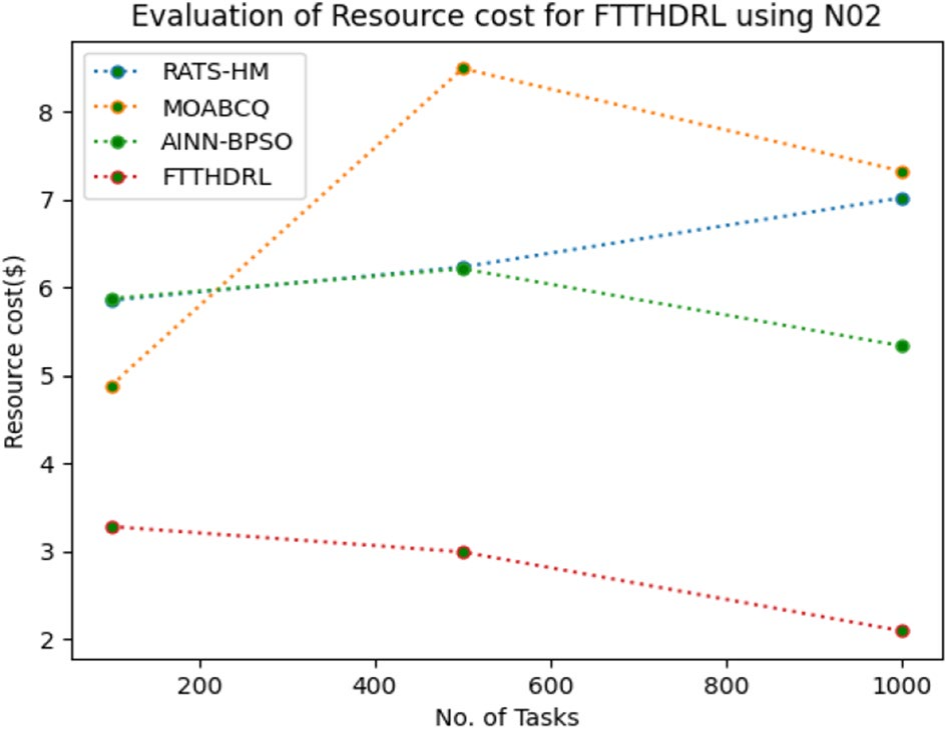
Resource cost using N02.

**Figure 35:**
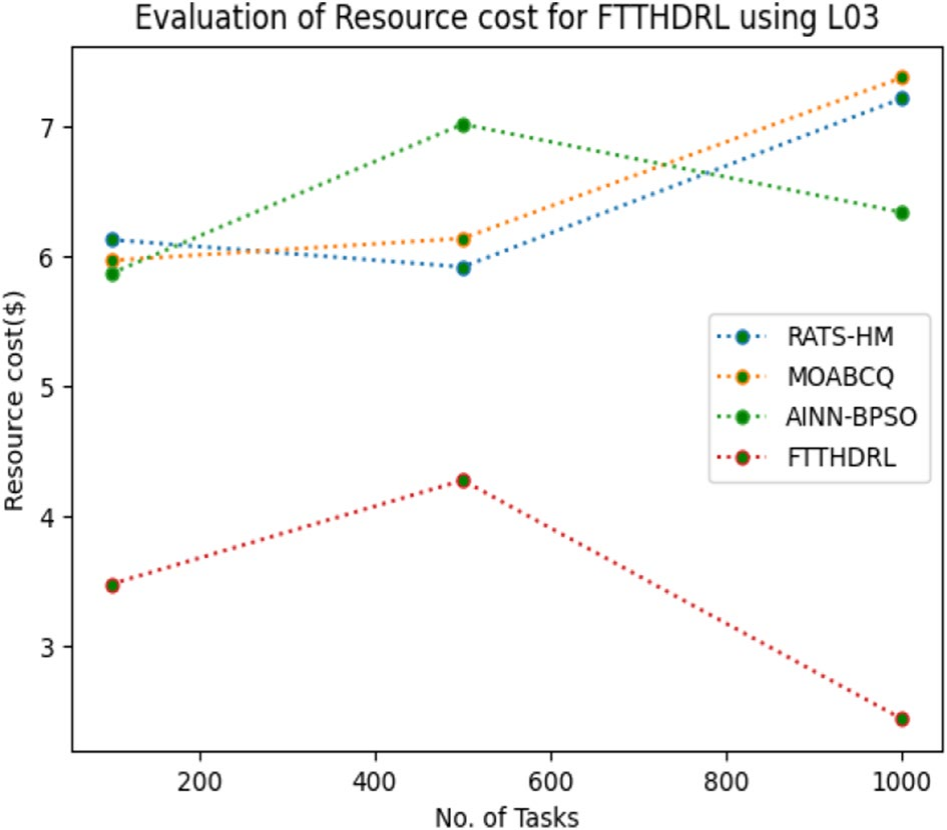
Resource cost using L03.

**Figure 36:**
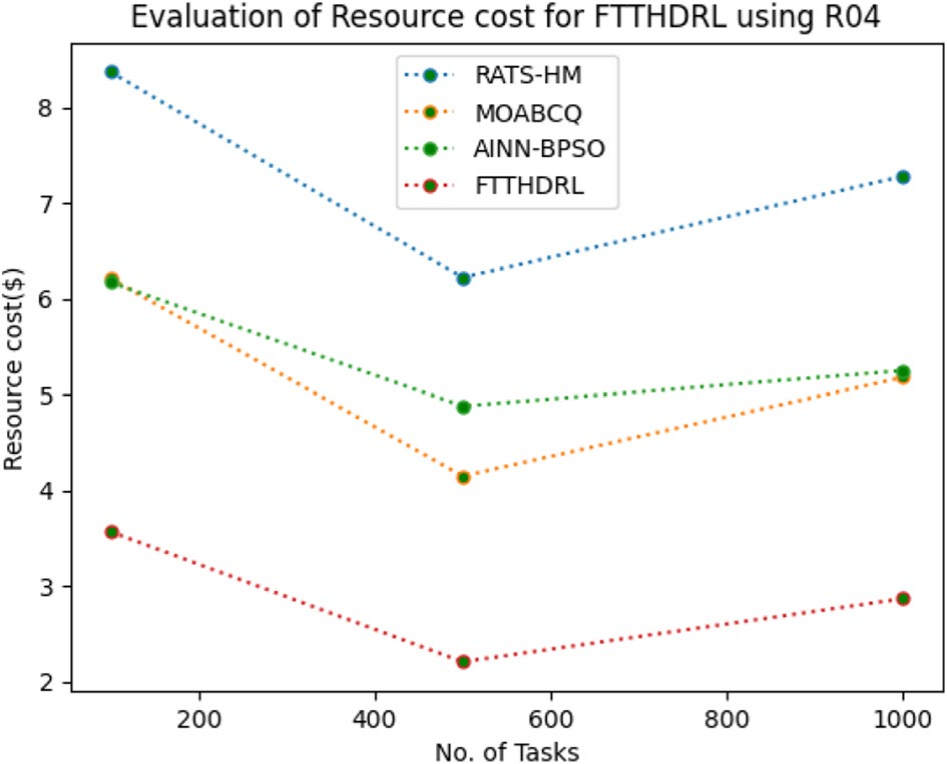
Resource cost using R04.

**Figure 37:**
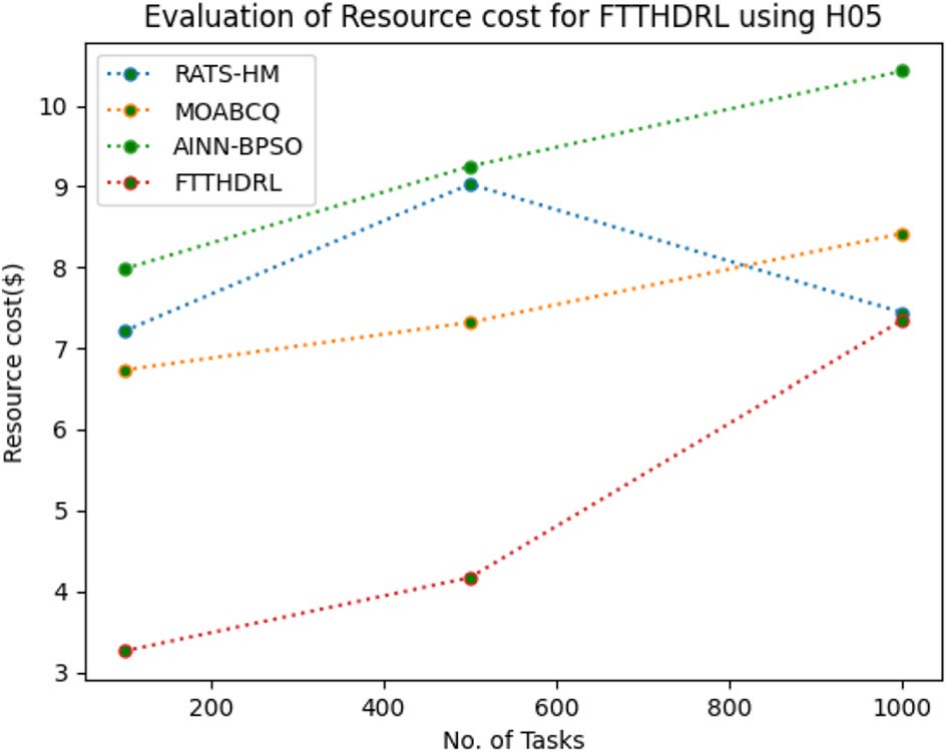
Resource cost using H05.

**Figure 38:**
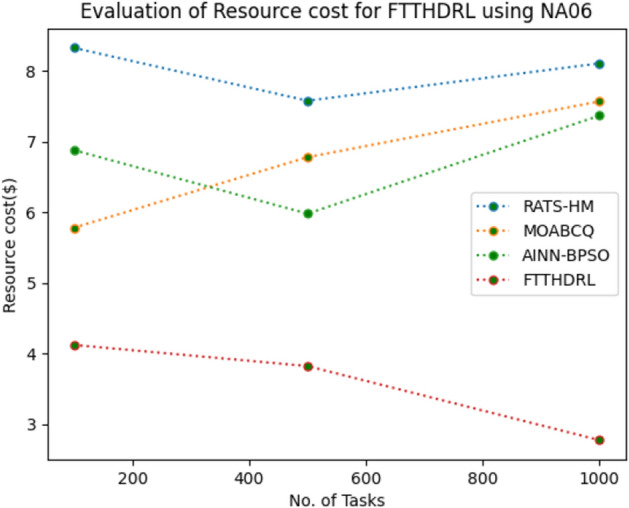
Resource cost using NA06.

In this Section “Evaluation of resource cost for FTTHDRL”, we evaluated Resource cost for our proposed FTTHDRL by giving input workload from various fabricated workloads from U01, N02, L03, R04 and real time parallel worklogs from H05, NA06. Proposed FTTHDRL compared over existing RATS-HM, MOABCQ, AINN-BPSO approaches. Table 9 represents Resource cost for 100, 500, 1000 tasks. Figures 33, 34, 35, 36, 37, 38 represents Resource cost for U01, N02, L03, R04, H05, NA06 respectively. After observing results generated Resource cost for FTTHDRL minimizes Resource cost greatly over state of art algorithms.

**Table 9:** Evaluation of resource cost for FTTHDRL.

Tasks	RATS-HM	MOABCQ	AINN-BPSO	FTTHDRL
100(U01)	5.92	6.76	4.97	4.05
500(U01)	7.14	7.12	6.57	3.92
1000(U01)	8.26	6.49	7.05	4.02
100(N02)	5.85	4.88	5.87	3.28
500(N02)	6.23	8.49	6.21	2.99
1000(N02)	7.02	7.32	5.33	2.09
100(L03)	6.13	5.97	5.87	3.48
500(L03)	5.92	6.14	7.02	4.28
1000(L03)	7.22	7.38	6.34	2.44
100(R04)	8.37	6.21	6.17	3.57
500(R04)	6.22	4.15	4.88	2.21
1000(R04)	7.28	5.18	5.25	2.87
100(H05)	7.21	6.73	7.98	3.26
500(H05)	9.03	7.32	9.25	4.17
1000(H05)	7.44	8.42	10.43	7.35
100(NA06)	8.33	5.78	6.88	4.12
500(NA06)	7.58	6.78	5.98	3.82
1000(NA06)	8.11	7.57	7.37	2.77

### Simulation analysis and discussion of results

Section “Simulation analysis and discussion of results” discusses generated result analysis and discussion about how they have improved over existing approaches. Initially, we fabricated different datasets indicated as U01, N02, L03, R04, H05, NA06. Generated results for proposed FTTHDRL evaluated over state of art approaches RATS-HM, MOABCQ, AINN-BPSO to check efficacy of our proposed algorithm. The below Table 10 represents makespan improvement for FTTHDRL, Table 11 represents improvement of rate of failures for FTTHDRL, Table 12 represents improvement of availability of VMs for FTTHDRL, Table 13 represents improvement of success rate of VMs for FTTHDRL, Table 14 represents improvement of turnaround efficiency of VMs for FTTHDRL, Table 15 represents minimization of resource cost for FTTHDRL. From these results, we can clearly observe that our proposed FTTHDRL dominates all state of art approaches for above specified parameters.

**Table 10:** makespan improvement for FTTHDRL over state of art algorithms.

Dataset	RATS-HM	MOABCQ	AINN-BPSO
U01	23.48	36.27	32.12
N02	24.76	26.88	28.08
L03	30.21	37.98	22.57
R04	16.88	25.22	11.98
H05	30.7	47.43	42.76
NA06	21.26	39.21	28.09

**Table 11:** Rate of failures improvement for FTTHDRL over state of art algorithms.

Dataset	RATS-HM	MOABCQ	AINN-BPSO
U01	62.76	64.57	54.75
N02	66.27	60.78	57.83
L03	68.47	61.24	32.57
R04	52.54	66.32	66.14
H05	71.53	71.29	69.31
NA06	72.11	74.53	71.12

**Table 12:** Availability of VMs improvement for FTTHDRL over state of art algorithms.

Dataset	RATS-HM	MOABCQ	AINN-BPSO
U01	23.48	32.17	26.78
N02	27.97	42.59	29.86
L03	18.78	39.67	25.87
R04	27.87	18.95	27.88
H05	37.25	39.78	43.08
NA06	72.06	46.32	34.38

**Table 13:** success rate of VMs improvement for FTTHDRL over state of art algorithms.

Dataset	RATS-HM	MOABCQ	AINN-BPSO
U01	47.24	24.73	40.54
N02	44.32	37.24	15.87
L03	39.67	49.36	16.88
R04	45.25	39.87	34.08
H05	71.16	33.17	40.76
NA06	64.54	42.12	71.44

**Table 14:** Turnaround efficiency of VMs improvement for FTTATS over state of art algorithms.

Dataset	RATS-HM	MOABCQ	AINN-BPSO
U01	42.76	51.21	50.08
N02	40.46	42.35	32.78
L03	41.28	34.21	30.13
R04	32.87	32.67	19.27
H05	68.56	48.12	40.34
NA06	38.57	40.12	20.36

**Table 15:** Resource cost improvement for FTTHDRL over state of art algorithms.

Dataset	RATS-HM	MOABCQ	AINN-BPSO
U01	20.24	31.54	30.65
N02	22.12	23.76	24.99
L03	31.08	32.67	23.21
R04	18.92	19.48	15.73
H05	21.03	19.08	17.45
NA06	20.04	22.17	25.18

## Conclusion and future works

Task scheduling is a crucial aspect in cloud computing paradigm as tasks arised from various resources and coming to cloud console need different processing capacities of virtual resources. In order to match task capacities with virtual resources an effective scheduler needed for cloud provider. Ineffective task scheduler leads to failures of tasks on VMs which doesn’t match task capacities to VM effectively. Thus, in this research to minimized failures of tasks and to match tasks appropriately to VMs we proposed a Fault tolerant trust aware task scheduler using Harris Hawk and Deep reinforcement based approach (FTTHDRL) in multi cloud environment. Initially we captured task, VM priorities to carefully map tasks to appropriate VMs. These priorities are fed to scheduler which is integrated with Harris Hawk optimization and DQN model which is a reinforcement learning approach which is a hybridized methodology used in our model. It generates schedules initially using Harris hawk algorithm and these generated schedules are optimized by DQN model to optimize parameters. Simulations are conducted on Cloudsim. For evaluating FTTHDRL, we used fabricated workload indicated as U01,N02, L03,R04. After this, we used realtime worklogs H05, NA06 used in simulation to evaluate FTTHDRL. Proposed FTTHDRL is evaluated over state of art approaches RATS-HM, AINN-BPSO, MOABCQ. From the observed results, FTTHDRL dominates existing algorithms by minimizing makespan, resource cost, rate of failures while improving trust based parameters. Shortcomings observed in our proposed research are it is not able to predict upcoming tasks thus, in future, we integrate a prediction module in the scheduler to predict tasks by using model to effectively schedule tasks in cloud paradigm.

## Data Availability

Researchers Supporting Project number (RSPD2023R576), King Saud University, Riyadh, Saudi Arabia.
